# PRRs-Dependent and Independent Mechanisms of STING Signaling in Inflammatory and Autoimmune Diseases

**DOI:** 10.3390/biomedicines13102533

**Published:** 2025-10-17

**Authors:** Le Xu, Jingrou Li, Xingchen Zhu, Liting Zhou, Zhirong Sun, Zhipeng Zhang, Wei Xu, Yahui Song

**Affiliations:** 1 Jiangsu Provincial Key Laboratory of Infection and Immunity, Institute of Biology and Medical Sciences, Soochow University, Building 703, 199 Ren-Ai Road, Suzhou 215123, China; 20224250016@stu.suda.edu.cn (L.X.);; 2 Center of Clinical Laboratory and Translational Medicine, The Fourth Affiliated Hospital of Soochow University (Suzhou Dushu Lake Hospital), Suzhou 215000, China

**Keywords:** STING, cGAS, signaling pathway, innate immunity, PRRs independent

## Abstract

The stimulator of interferon genes (STING) serves as a pivotal signaling hub in innate immunity, orchestrating type I interferon (IFN-I) and pro-inflammatory responses upon detection of cytosolic DNA. While the canonical cyclic GMP-AMP synthase (cGAS)-STING axis has been extensively studied in host defense and sterile inflammation, increasing evidence indicates that STING can also be activated through a variety of both pattern recognition receptors (PRRs)-dependent and PRRs-independent mechanisms. In this review, we comprehensively summarize the molecular pathways through which PRRs—including cGAS, interferon gamma inducible protein 16 (IFI16), DEAD-box helicase 41 (DDX41), and DNA-dependent protein kinase (DNA-PK)—engage and regulate STING activation. Beyond PRRs-triggered pathways, we explore emerging evidence of PRRs-independent STING activation, driven by genetic mutations, endoplasmic reticulum (ER) stress, dysregulated intracellular trafficking, and impaired protein degradation. These mechanisms contribute to the pathogenesis of a broad spectrum of inflammatory and autoimmune disorders affecting multiple organ systems, including the digestive, cardiovascular, renal, pulmonary, and nervous systems. We also highlight the current landscape of pharmacological inhibitors targeting cGAS and STING, categorized according to their mechanisms of action and therapeutic potential. The redundancy and complexity of components within the STING signaling network present challenges in effectively suppressing inflammatory overactivation by targeting a single molecule. Nevertheless, the central role of STING offers multiple opportunities for therapeutic intervention, whether by modulating upstream or downstream signaling elements. This review not only provides a systematic framework for understanding the intricacies of STING signaling, but offers insights into the development of next-generation therapeutics aimed at selectively modulating STING activity in disease contexts.

## 1. Introduction

The mammalian immune system protects the body from infections and maintains tissue homeostasis [1]. It comprises both innate and adaptive branches [2]. Innate immunity serves as the first line of defense by utilizing pattern recognition receptors (PRRs) to detect pathogen-associated molecular patterns (PAMPs) and damage-associated molecular patterns (DAMPs), while adaptive immunity provides tailored protection that is enhanced upon re-exposure [3,4]. Nucleic acids, essential for pathogen parasitism, are recognized through evolutionarily conserved biological mechanisms. Pathogenic nucleic acids within endosomes are detected by Toll-like receptors (TLRs), whereas cytosolic nucleic acids are primarily sensed by receptors such as retinoic acid-inducible gene I (RIG-I)-like receptors (RLRs), cGAS, and absent in melanoma 2 (AIM2) [5]. Among these, cGAS is a key component of the DNA-sensing pathway, playing an essential role in initiating innate immune responses to infections and cellular damage. Notably, recognition of self-DNA can lead to aberrant innate immune activation, contributing to disease pathogenesis [6]. STING acts as a critical adaptor protein that binds directly to 2′3′-cyclic GMP-AMP (cGAMP), which is synthesized by cGAS, thereby linking DNA sensors to downstream innate immune signaling pathways [7]. Although cyclic dinucleotides (CDNs), such as cGAMP, are classical activators of STING, several other DNA sensors that do not produce CDNs—such as interferon gamma-inducible protein 16 (IFI16) [8], DDX41 [9], DNA-PK [10], heterogeneous nuclear ribonucleoprotein A2/B1 (hnRNPA2B1) [11]—have also been shown to activate STING.

The activation of downstream signaling pathways by STING is a complex biological process involving its post-translational modifications, conformational changes, dimerization [12], transport between subcellular compartments, and the formation of protein complexes [13]. The primary goal of PRRs-mediated STING activation is to ensure that these downstream events occur in a tightly regulated manner. Notably, disruption of STING homeostasis under physiological conditions can also lead to the activation of STING-mediated signaling pathways. Emerging evidence suggests that cellular homeostatic regulation of STING, rather than upstream DNA sensing alone, plays a more decisive role in modulating STING signaling. These regulatory mechanisms include redox balance [14], STING mutation [15], STING autoinhibition in ER [16], STING trafficking [17], and linear ubiquitination of STING [18], among others. Although some studies have not conclusively demonstrated that disturbance of STING homeostasis under normal conditions directly activates downstream signaling, we propose that non-PRRs-mediated STING activation is of considerable importance, and the possibility of direct activation should not be excluded.

Aberrant activation or suppression of the STING signaling pathway has been implicated in a wide range of diseases, including autoimmune, cardiovascular, and neurodegenerative disorders. To provide a clearer mechanistic perspective, this review outlines STING signaling with an emphasis on PRRs dependence, contrasting canonical (e.g., DNA-triggered) and non-canonical activation mechanisms. We further highlight the distinct and system-level roles of these two activation modes across organ systems and outlines a systematic framework that differentiates infection-driven, self-limiting responses from persistent non-PRRs dysregulation in chronic disease. Moreover, we categorize inhibitors according to the activation mode they target and underscore the potential of precision and multi-target strategies as promising therapeutic directions.

Unlike previous reviews that have primarily concentrated on the canonical cGAS-DNA-STING axis or on the development of agonists in cancer immunotherapy [19,20], our work provides a comprehensive, system-level synthesis of both PRRs-dependent and PRRs-independent mechanisms of STING activation. Although a few publications have touched on PRRs-independent activation, they often lack a coherent framework linking these mechanisms to disease pathogenesis and therapeutic strategies [21]. By contrast, this review summarizes a systematic framework that integrates diverse activation modes with organ-specific disease contexts and highlights their implications for precision therapeutic development. By integrating evidence across cardiovascular, renal, pulmonary, digestive, neurological, and autoimmune diseases, we delineate the underappreciated contribution of non-PRRs triggers—including STING mutations, ER stress, and trafficking or degradation defects—that remain overlooked in the current literature. This dual focus allows us to propose a mechanism-based classification of therapeutic inhibitors, linking activation mode to clinical context. Furthermore, we discuss translational challenges such as species-specific differences, toxicity, and off-target risks, and introduce forward-looking perspectives, including nanoparticle-based delivery systems and combination strategies. Collectively, this review advances the field by offering not only a critical summary of existing knowledge but also a novel framework and therapeutic outlook that extend beyond the prevailing DNA-centric paradigm.

## 2. PRRs Dependent STING Signaling

### 2.1. DNA-cGAS-STING Signaling

**Figure 1:**
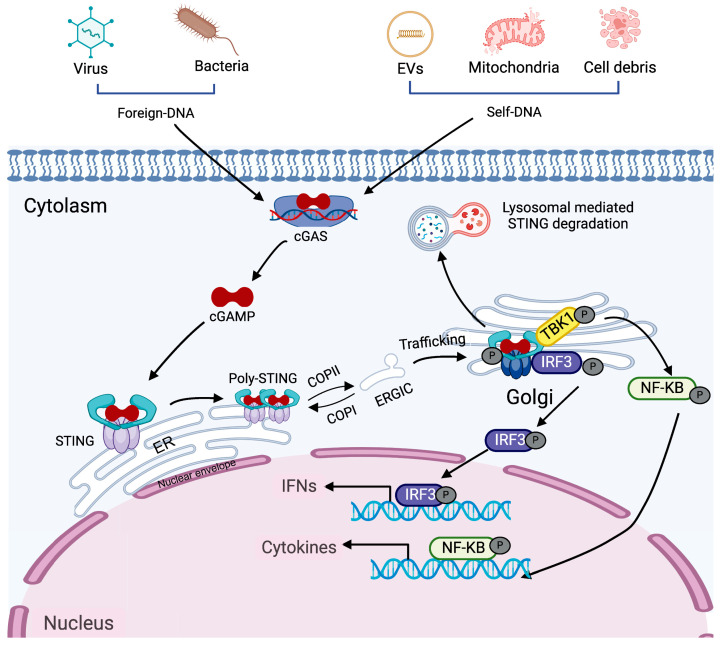
Signaling mechanism of the cGAS dependent STING pathway. dsDNA, originating from viruses, bacteria, parasites, or self-derived sources such as extracellular vesicles, mitochondria, and cellular debris, enters the cytoplasm and is detected by the sensor cGAS. This recognition leads to the synthesis of cGAMP. cGAMP subsequently binds to STING in the ER, inducing a conformational change. This conformational change takes place in the linker region, causing the ligand-binding domain to rotate into a parallel alignment with the transmembrane domain, thereby promoting STING activation and its movement from the ER to the ERGIC vesicles. Once STING reaches the Golgi, the CTT of dimerized STING recruits TBK1. This process phosphorylates STING. Furthermore, the CTT acts as a binding site for IRF3, enhancing its phosphorylation. Once phosphorylated, IRF3 forms dimers and moves into the nucleus to drive the expression of interferons (IFN). Additionally, STING activates the nuclear factor kappa B essential modulator (NEMO)-IKKα/β pathway, which further stimulates TBK1/IKK activation and boosts the transcription of pro-inflammatory cytokines and chemokines. Concurrently, the phosphorylation of STING in the Golgi marks the initiation of lysosome-mediated degradation.

The classical mode of STING activation relies on PRRs upon recognizing foreign or self-DNA, serving as a key mediator in the innate immune defense against microbial pathogens and autoimmune disorders. cGAS is the main endogenous classical DNA sensor that activates STING [22,23]. This process, illustrated in Figure 1 and referred to as the classical DNA-cGAS-STING pathway, is mainly involved in detecting and restricting the spread of cytosolic foreign DNA. Binding of double stranded DNA (dsDNA) activates the enzymatic function of cGAS, leading to the production of cGAMP, a secondary messenger that conveys the danger signal. cGAMP subsequently interacts with STING located on the endoplasmic reticulum membrane, triggering the release of STING’s C-terminal tail (CTT) and facilitating the polymerization of its dimers [24]. This conformational shift transforms STING from an inactive “open” state to an active “closed” form. Polymerized STING is subsequently trafficked from the ER to the ER–Golgi intermediate compartment (ERGIC) and ultimately to the Golgi apparatus, where the CTT recruits TANK-binding kinase 1 (TBK1) [25]. STING polymerization facilitates TBK1 activation via trans-autophosphorylation. Activated TBK1 then phosphorylates the C-terminal region of STING, enabling recruitment of interferon regulatory factor 3 (IRF3), which then undergoes phosphorylation and dimerization [26]. The IRF3 dimers translocate into the nucleus to initiate IFN-I gene transcription, propagating the antiviral response in an autocrine and paracrine manner [27]. Secreted IFN-I binds to its receptors, interferon alpha/beta receptor 1 (IFNAR1) and IFNAR2, initiating the recruitment of Janus kinase 1 (JAK1) and tyrosine kinase 2 (TYK2). These kinases phosphorylate signal transducer and activator of transcription (STAT) proteins. Phosphorylated STAT1 and STAT2 form a complex with interferon regulatory factor 9 (IRF9) which translocates to the nucleus to induce the expression of interferon-stimulated genes (ISGs) [28]. In parallel with IRF3 activation, STING associates with IκB kinase (IKK), facilitating the induction of genes encoding pro-inflammatory cytokines and chemokines [29]. Following signal termination, STING is degraded via the lysosomal or autophagosomal pathways to prevent sustained immune activation and maintain immune homeostasis.

In addition to recognizing pathogen-associated molecular patterns, cGAS can detect both foreign and self-DNA, including microbial DNA, mitochondrial DNA, and DNA released from dying tumor cells [30,31]. Its activation is not dependent on specific DNA sequences, but rather on DNA length—longer DNA strands more effectively activate cGAS and promote its phase separation into liquid-like droplets [32]. Phase separation of cGAS is governed by its local concentration along with that of DNA, implying that sufficient DNA levels are essential to initiate robust activation [24,32].

### 2.2. DNA-IFI16-STING Signaling

In 2010, Andrew G. Bowie’s team identified IFI16 as a DNA sensor [8], subsequent research by Martin Roelsgaard Jakobsen revealed its regulatory role in the cGAS-STING pathway [33]. While early studies primarily focused on the IFI16-mediated regulation within this pathway, accumulating evidence suggests that IFI16 can also activate STING independently of cGAS [34,35].

In the canonical cGAS-STING pathway, cGAMP binding induces STING phosphorylation and dimerization, which process modulated by IFI16. Notably, IFI16 can trigger IFN-I production even in the absence of cGAS, although the response is less robust [33]. Further supporting cGAS-independent STING activation, extracellular exposure of mitochondria does not generate 2′3′-cGAMP, and inhibition of cGAS fails to prevent endothelial cell activation [35]. Mechanistic insights from Leonie Unterholzner’s work demonstrate that damaged DNA promotes the formation of a STING-IFI16-p53-RAF6 complex via AIM2, thereby activating both IRF3 and NF-κB signaling pathways [36]. Critically, IFI16-dependent STING activation has been implicated in diverse pathologies, including cervical cancer [37], intestinal Behçet’s syndrome [38], melanoma [39], lung adenocarcinoma [40].

### 2.3. DNA-(DNA-PK)-STING Signaling

Activation of the DNA damage response (DDR) involves phosphoinositide-3-kinase-related kinase (PIKK) family kinases, including DNA-PK, ataxia-telangiectasia mutated (ATM), and ATM-and Rad3-related protein (ATR), which catalyze phosphorylation of downstream effector proteins [41]. DNA-PK is a multi-component complex composed of a catalytic subunit (DNA-PKcs), the Ku70/80 heterodimer, and DNA ends. In 2012, Ferguson et al. reported that DNA-PK contributes to the activation of innate immune responses [10]. Accumulating evidence indicates that DNA-PK complex regulates multiple pathways triggered by intracellular DNA sensing, with specificity varying according to cellular context. In human fibroblasts, cGAS, STING, and DNA-PK collectively play crucial roles in the induction of IFN-I [42]. In monocytic cells, Ku proteins which is identified as cGAS-interacting partners significantly enhance the DNA-binding affinity of cGAS [43]. DNA-PK can activate antiviral responses via a STING-independent mechanism in human cells [44]. These findings suggest that DNA-PK may trigger IRF3 activation through non-canonical pathway independent of the canonical cGAS-STING axis. Although direct evidence of DNA-PK-mediated STING activation remain elusive, the possibility cannot be excluded. cGAS-independent IFN-I induction by DNA-PK may represent species-specific, potentially account for the limited number of related studies to date.

### 2.4. DNA-DDX41-STING Signaling

Before the identification of cGAS as the primary DNA sensor that activates STING, DDX41, together with IFI16, had been recognized as a key DNA sensor involved in triggering STING signaling [9]. Subsequent studies revealed that DDX41 can recognize both DNA and CDNs [45], with structural and biochemical analyses further elucidating the molecular basis underlying its recognition of dual PAMPs [46]. Recent evidence indicates that DDX41 can directly activate STING [47] as well as modulate the cGAS-STING pathway [48]. This functional versatility within the STING network suggests DDX41 may utilize previously uncharacterized mechanisms for STING activation.

In addition to canonical DNA sensors such as cGAS, IFI16, and DNA-PK, other PRRs, including TLRs and AIM2, have also been implicated in modulating STING signaling. Although TLRs are primarily localized within endosomal compartments and typically signal through myeloid differentiation primary response 88 (MyD88)-or TIR-domain-containing adapter-inducing interferon-β (TRIF)-dependent pathways, increasing evidence indicates that they can indirectly influence STING activity. This regulation may occur through mechanisms such as cytokine production, modulation of STING expression, or alterations in the intracellular signaling environment. For instance, TLR3 and TLR9 have been shown to either enhance or suppress STING-mediated responses, depending on the cell type and nature of the immune stimulus [49]. In contrast, AIM2 appears to negatively regulate STING signaling, possibly by sequestering cytosolic DNA or competitively interacting with other DNA sensors [50]. Beyond these well-established receptors, emerging studies have begun to identify additional PRRs including certain RNA helicases and nucleotide-binding and oligomerization domain (NOD)-like receptors (NLRs), that may interface with the STING pathway either directly or via adaptor proteins. For instance, NLRP3 [51] and NLRC4 [52] cooperates with STING to amplify IFN I, whereas NLRC3 acts as a negative regulator by disrupting the STING-TBK1 interaction [53]. However, the precise mechanisms of crosstalk between STING and NLRs remain largely undefined. Collectively, these findings highlight the complexity and redundancy of PRRs-mediated STING regulation. They also underscore the need for further investigation into the hierarchical organization, context-dependent functions, and in vivo relevance of these signaling interactions, which is crucial for the progression of STING-targeted immunotherapy development.

## 3. PRRs-Independent STING Signaling

**Figure 2:**
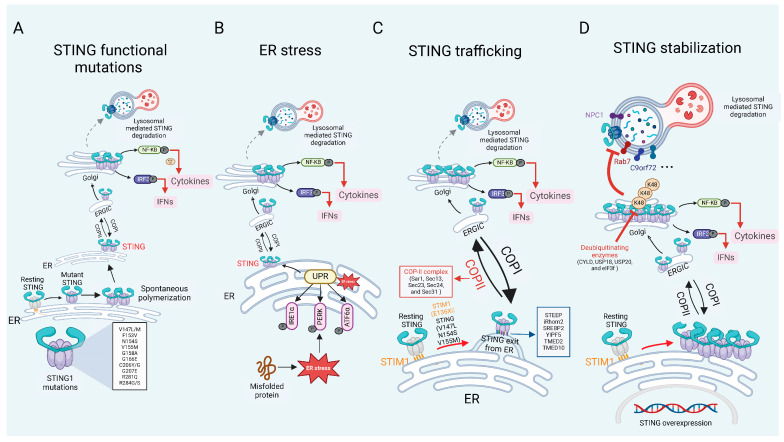
PRRs independent STING signaling in inflammatory and autoimmune diseases. (**A**) Gain-of-function mutations in STING1 cause persistent STING activation, resulting in continuous production of IFN I and cytokines. (**B**) As an ER protein, STING can detect ER stress, leading to its spontaneous activation. (**C**) STING is localized in the ER and is stabilized by resident ER proteins, STIM1 and TOLLIP. Mutations or deletions of STIM1 and TOLLIP result in the sustained transport of STING to the Golgi apparatus. Beyond these proteins, additional factors such as ER exit proteins and COP-associated proteins are essential for mediating the transport of STING from the ER to the Golgi, and they also contribute to the prolonged activation of STING. (**D**) Under normal circumstances, STING is degraded to prevent an inflammatory storm caused by its excessive activation. However, specific proteins can inhibit STING degradation through mechanisms such as deubiquitination or lysosomal blockade, leading to its overexpression and sustained activation.

While the mechanisms of PRRs-dependent STING activation have been extensively characterized, our understanding of PRRs-independent STING activation remains limited. However, the accumulating evidence indicates that functional mutations, spontaneous oligomerization, sustained trafficking, and enhanced protein stability can trigger ligand-independent STING activation. In this section, we highlight recent advances in this area and discuss how PRRs-independent STING signaling is activated (Figure 2), as well as how key regulatory factors of STING are involved, although some of these factors currently lack evidence supporting their ability to activate STING independently (Table 1). We focus on four key aspects: (1) STING1 mutations. Specific mutations within the STING1 gene result in constitutive activation of the STING pathway, thereby driving chronic inflammatory responses and the development of autoimmune diseases, as exemplified by STING-associated vasculopathy with onset in infancy (SAVI). [15]. (2) ER stress. The ER serves as the initial site for STING post-translational modifications and is a critical hub for regulating STING signaling [54,55]. (3) Dysregulated Trafficking. Aberrant or prolonged trafficking of STING from the ER to the Golgi apparatus can result in its persistent activation, even in the absence of PRRs signaling [56,57]. (4) STING Stabilization. Enhanced stability of the STING protein, due to impaired degradation or defective signal termination, can also lead to sustained pathway activation [58].

### 3.1. STING Mutations

Gain-of-function mutations in STING induce spontaneous polymerization and trafficking to the ERGIC, resulting in STING activation independent of cGAMP and causing persistent production of IFN I. These mutations are associated with a severe autoinflammatory disorder known as SAVI [59,60]. SAVI mutations occur in key regions of the STING, including dimerization domain: mutations such as V147L, V147M, F153V, V155M, N154S, G158A, F269S and G166E [15,61,62,63,64], ligand-binding domain: mutations C206Y/G and G207E. and polymerization interface: mutations including C206Y/G, R281Q, and R284G/S [65]. These mutations lead to the autonomous overproduction of IFN I, occurring independently of cGAS binding or activation by 2′3′-cGAMP. The hyperactivation of STING may result from increased dimerization or polymerization [66], reduced autoinhibition of STING oligomerization [16], spontaneous trafficking, or destabilization of the ligand-binding domain [67]. The aberrant activation driven by STING mutations essentially stems from residue alterations that weaken negative regulation while exaggerating positive regulation of STING. In 2014, Liu et al. reported six children showed signs of systemic inflammation, interstitial lung disease (ILD), and skin vasculopathy. Among them, four patients exhibited a marked interferon-stimulated gene (ISG) signature in their peripheral blood specimens [68]. The same year, Jeremiah et al. reported a family with pulmonary fibrosis, recurrent fevers, and autoimmunity, with genetic analysis revealing the presence of the V155M STING mutation in four pediatric patients [63]. Since then, over 70 SAVI cases have been documented, with clinical phenotypes expanding beyond neurologic and cutaneous manifestations to include arthritis, thyroiditis [69], glomerulonephritis [70], cerebrovascular involvement [71], and other organ pathologies. In mice, knock-in mutations such as N153S or V154M replicate SAVI, gives rise to pulmonary disorders, cytokine secretion, cutaneous ulcerations, and premature mortality [59,60,72].

### 3.2. ER Homeostasis

STING, as an ER-resident protein, is closely related to ER stress, with increasing evidence suggesting that ER homeostasis directly or indirectly affects STING activation. In various disease contexts, ER stress has been shown to modulate STING activity and drive inflammation [55,73,74]. ER is involved in the synthesis and modification of STING, serving as the site for ligand binding and dimerization. The regulation of ER-related processes modulates innate immunity by controlling the size of the STING pool [55]. The homeostasis of the ER is intricately linked to the activation of STING. When unfolded or misfolded proteins accumulate in the ER lumen, ER stress is triggered [75]. The role of ER homeostasis in the function of STING has been extensively discussed in another review [54]. ER homeostasis not only directly activates immune responses but also plays a role in various pathological conditions, including inflammatory, tumorigenic, neurodegenerative, and metabolic diseases [76] such as cardiac hypertrophy [74,77,78], traumatic brain injury [79], myocardial ischemia/reperfusion injury [77], and diabetic retinopathy [78,80]. Under these conditions, ER stress amplifies STING signaling, resulting in heightened inflammation and tissue damage.

### 3.3. STING Trafficking

Membrane exchange between organelles, primarily mediated by vesicular trafficking, is critical for cellular homeostasis. As a protein localized on the ER membrane, STING depends on trafficking process for its activation. The movement of STING from the ER to the Golgi apparatus is a crucial step that indicates the activation of STING signaling. Under normal conditions, STING associates with ER retention proteins to keep it in an inactive state, preventing premature immune responses. However, the deficiency or mutation of these retention factors can release STING, leading to aberrant STING activation and subsequent autoimmune diseases [81].

Stromal interaction molecule 1 (STIM1), an ER-resident protein that senses ER Ca^2+^ levels, binds to STING to retain it within the ER, thereby preventing its transport and activation. However, a lack of STIM1 increases the production of type I IFNs through a mechanism that depends on STING, and mutations in STIM1 (such as E136X) and STING (including V147L, N154S, and V155M) impair this interaction, leading to increased trafficking of STING to ERGIC, which is associated with autoimmune complications [82,83]. On the other hand, STING ER exit protein (STEEP) mediates STING exit from the ER. STEEP facilitates STING exit from the ER by inducing phosphatidylinositol-3-phosphate (PtdIns(3)P) production, which increases ER membrane curvature and promotes COP II-mediated vesicle formation. In the pathological setting of SAVI, STING variants exhibit enhanced binding to STEEP, leading to excessive PtdIns(3)P accumulation, aberrant ER exit of STING, and persistent type I IFN signaling even in the absence of cGAMP stimulation. This mechanism emphasizes the therapeutic potential of targeting STEEP-STING interactions to manage STING-driven inflammatory disorders such as SAVI and systemic lupus erythematosus (SLE) [84]. Additionally, inactive rhomboid protein 2 (iRhom2) serves functioning as a promoter of DNA virus-triggered IFN I production by recruiting TRAPβ to the STING complex, which assists in the transport of STING from the ER to the Golgi, thereby regulating STING activation [85]. Similarly, sterol regulatory element-binding protein 2 (SREBP2) mediates STING triggering through its trafficking, presenting a noncanonical activation pathway that may impact neuropathology and could provide a potential treatment target for Niemann-Pick type C disease [86].

The coat protein complex II (COP II), whose core components include Sar1, Sec13, Sec23, Sec24, and Sec31, mediates vesicular transport from the endoplasmic reticulum to the Golgi apparatus and is indispensable for STING trafficking. Knockdown of COP II proteins experiment confirms the importance of COP II-mediated transport in STING signaling. Additionally, SREBP cleavage-activating protein (SCAP), a cholesterol sensor, recruits STING to Golgi in macrophages, initiating NF-κB and playing a role in non-alcoholic fatty liver disease (NAFLD) [87]. Acyl-CoA binding domain protein 3 (ACBD3), a Golgi-resident protein, also facilitates the movement of STING from ER to Golgi. Deletion of ACBD3 disrupts STING vesicular trafficking and type I IFNs responses, thus revealing its role in a nonclassical cargo-concentrating system that drives STING ER exit [88,89]. Similarly, retrograde transport is essential for silencing STING signaling, and impairments in this pathway are implicated in coatomer subunit α (COPA) syndrome, a disorder marked by autoinflammatory and autoimmune manifestations including alveolar bleeding, arthritis, and nephritis. COPA syndrome arises from mutations in the COPA gene, which encodes the α-subunit of the coatomer protein complex I (COP I). These mutations impair COP I-mediated retrograde transport, preventing STING from returning to the ER from the Golgi, ultimately resulting in the STING accumulation in Golgi apparatus and its subsequent activation within the trans-Golgi network, triggering chronic inflammation [90,91]. In summary, STING’s ER-Golgi translocation is tightly regulated by multiple factors, and its dysregulation, whether through mutations or transport defects, can result in aberrant immune responses and autoimmune diseases. Understanding these mechanisms opens avenues for therapeutic interventions targeting STING trafficking in inflammatory diseases like SAVI, SLE, and COPA syndrome.

### 3.4. STING Stabilization

Overexpression of STING induces excessive immune activation and inflammation, which can cause tissue damage and harm the host. Therefore, tight regulation of STING expression is essential to prevent chronic inflammatory responses. This regulation is primarily achieved through two degradation pathways: degradation mediated by the ubiquitin-proteasome system and lysosomal degradation via trafficking pathways. Deubiquitinating enzymes such as cylindromatosis lysine 63 deubiquitinase (CYLD), ubiquitin-specific peptidase 18 (USP18), ubiquitin-specific peptidase 18 (USP20), and eukaryotic translation initiation factor 3 subunit F (eIF3f) remove K48-linked polyubiquitin chains from STING, thereby preventing proteasomal degradation and prolonging STING activity [56,92,93]. Lysosomal degradation constitutes another critical mechanism for limiting STING signaling. Inhibition of this pathway can lead to ligand-independent STING activation, promoting the development of inflammatory diseases. Toll-interacting protein (TOLLIP) stabilizes STING by directly binding to it and inhibiting degradation via the IRE1α and lysosomal pathways. Loss of TOLLIP results in enhanced STING degradation due to hyperactivation of the ER stress sensor IRE1α. In Huntington’s disease, polyQ-rich proteins in striatal neurons compete with STING for TOLLIP binding, impairing this interaction and reducing STING stability, ultimately dampening immune responses [58]. The Niemann-Pick type C1 (NPC1) protein is also essential for lysosomal trafficking of STING. NPC1 binds STING, recruits it to lysosomes, and blocks STING interaction with SREBP2. In *Npc1*^−/−^ mice, hyperactivation of STING leads to severe neurological damage, which can be significantly ameliorated by STING deficiency, resulting in improved motor function and reduced neuronal injury [86]. Recent studies have further identified the endosomal sorting complexes required for transport (ESCRT) pathway as a major route for STING degradation. The ESCRT machinery detects ubiquitinated STING on vesicles, facilitates its encapsulation into lysosomal associated membrane protein 1 (LAMP1)-positive compartments, and promotes degradation within lysosomes [94,95,96]. Collectively, these results highlight the critical role of precisely regulated STING degradation—via both proteasomal and lysosomal pathways—for maintaining immune homeostasis and preventing inflammatory or neurodegenerative diseases. Elucidating these mechanisms offers promising therapeutic targets for disorders associated with STING overactivation.

### 3.5. STING Functions as a More Central Node than PRRs

Increasing evidence suggests that PRRs-independent activation of STING deserves greater attention, as such conditions are frequently associated with severe clinical phenotypes and urgent unmet needs. For example, SAVI is a devastating autoinflammatory disease for which no effective therapy currently exists [68,97,98]. Similarly, in SLE and COPA syndrome, excessive STING activation can occur independently of cGAS through abnormal trafficking, post-translational modifications, or metabolic stress, leading to persistent type I interferon production [81,91,99,100]. Increasingly, STING regulators are found to act beyond modulation by directly activating STING without cGAS. We recommend evaluating them in cGAS-deficient settings to clarify their principal cGAS-independent functions.

Moreover, various forms of cell death, including apoptosis [101], necroptosis [102,103,104], and pyroptosis [105], ferroptosis [106] are related with self-DNA leakage or organelles damage triggering STING activation without the involvement of classical PRRs. These pathways highlight the ability of STING to act as a sensor of cellular stress and damage, integrating signals that extend beyond pathogen recognition.

In these contexts, targeting upstream PRRs such as cGAS would be insufficient, as pathological STING activation bypasses canonical ligand recognition. By contrast, STING remains the central hub, converging multiple upstream signals—both PRRs-dependent and independent—into a unified downstream cascade that induces type I interferons and inflammatory mediators. Consequently, direct inhibition of STING provides a broader and more universal therapeutic strategy, capable of addressing a wide spectrum of inflammatory and autoimmune disorders.

**Table 1:** PRRs-independent regulation of STING activation.

Activation Mode	Key Regulators	Mechanism	Disease Systems & Models	Reference
STING mutations	V155M, N154S, V147L, C206Y, G207E, R281Q, R284S, H72N, G166E site of STING	Mutation altering amino acids at the dimerization interface or ligand-binding domain.	SAVI associated disease	[61,62]
STING mutations	C88Y, R96L, V193I, C88Y, R96L, V193I	Mutations in the transmembrane domain alter STING topology and cause constitutive activation.	SAVI associated disease	[107]
ER stress	TOLLIP (positive regulator)	Transmembrane domain mutations disrupt STING topology, leading to constitutive activation.	Autoinflammatory disease	[58]
ER stress	HRD1 (negative regulator)	ERAD directly interacts with, and ubiquitinates, ER-resident resting STING, leading to its proteasomal degradation.	Viral infection and tumor models	[55]
ER stress	UNC13D (negative regulator)	UNC13D colocalizes with STING on the ER, inhibiting STING oligomerization and activation.	Cell level	[108]
STING trafficking	STIM1, NSP2, ISD017 (negative regulator)	STIM1 binds resting STING, retaining it on the ER and suppressing spontaneous activation; NSP2 and ISD017 further potentiate this STIM1-mediated inhibition.	Autoinflammatory disease	[82,83,109]
STING trafficking	STEEP (positive regulator)	Inducing COPII-mediated ER to Golgi trafficking of STING.	SAVI associated disease	[84]
STING trafficking	iRhom2, ADAM17 (positive regulator)	ADAM17 selectively stabilizes iRhom2 as a positive regulator of STING.	Viral infection and tumor models	[56,85]
STING trafficking	SCAP (positive regulator)	SCAP facilitates STING translocation to the Golgi.	nonalcoholic fatty liver disease	[87]
STING trafficking	COP II (positive regulator)	STING phosphorylation cues COPII transport; post-fusion coat loss at the ERGIC/cis-Golgi enables IRF3 recruitment for TBK1-mediated phosphorylation.	SAVI associated disease	[110]
STING trafficking	COP I (negative regulator)	Mutations in COP components result in aberrant ER localization of STING.	COPA syndrome	[81,91]
STING trafficking	ACBD3 (positive regulator)	ACBD3 facilitates COP II-independent ER exit of STING and its trafficking to the Golgi.	Cell level	[88]
STING trafficking	YIPF5 (positive regulator)	YIPF5 promotes STING signaling by recruiting it to COPII vesicles and driving ER-to-Golgi trafficking.	Viral infection	[111]
STING stabilization	CYLD, USP18, USP20, eIF3f (negative regulator)	STING stability is regulated by ubiquitination, and lysosomal degradation mechanisms.	Viral infection	[56,92,93]
STING stabilization	TOLLIP (positive regulator)	TOLLIP inhibits the lysosomal degradation of STING.	Huntington’s disease & viral infection	[58,112]
	IRE1α (negative regulator)	IRE1α positively regulates the lysosomal degradation of STING.	Huntington’s disease	[58,113]
STING stabilization	NPC1	NPC1 interacts with STING and recruits it to the lysosome for degradation in both human and mouse cells.	Niemann-Pick disease type C	[86]
STING stabilization	ESCRT	The ESCRT complex facilitates ubiquitin-mediated degradation of STING.	Cell level	[95,96]

## 4. STING Activation in Inflammatory and Autoimmune Diseases

PAMPs and DAMPs overlap substantially in recognition and signaling pathways but also retain independence [3]. Their receptors (TLRs, RLRs, NLRs, CLRs, ALRs, cytosolic DNA sensors) converge on downstream cascades, including NF-κB, MAPK, IRF3/7 activation, and inflammasome assembly, which drive cytokine and interferon production [114,115]. Notably, cGAS can respond to non-self DNA, such as DNA from DNA viruses or retroviruses [116], intracellular bacterial DNA (PAMPs), and can also be activated by self-DNA such as extracellular, mitochondrial, and nuclear DNA that can enter the cytoplasm (DAMPs) [100,117]. Accordingly, STING activation arises in two distinct contexts: infection-related inflammation and infection-independent autoimmunity or autoinflammation [118]. In infection-related settings, STING serves as a host defense mechanism, sensing cytoplasmic DNA and triggering a robust, self-limiting “sacrificial” inflammatory response that, despite collateral tissue injury, ensures rapid immune cell recruitment and pathogen clearance [119,120]. Under normal conditions, anti-inflammatory mediators restrain this response once pathogen burden is controlled. However, when pathogens cannot be completely eliminated, or when the resolution mechanisms that normally terminate inflammation are insufficient, STING signaling may remain aberrantly active, leading to sustained inflammation and tissue injury [68]. In contrast, infection-independent STING activation occurs without external pathogens and is driven by intrinsic abnormalities such as nucleic acid clearance defects (e.g., TREX1 [121,122] or DNase deficiencies [123]), gain-of-function STING mutations (e.g., SAVI [68,97]), ER stress [124], or dysregulated trafficking. Inflammation becomes decoupled from host defense against pathogens, resulting in persistent and maladaptive IFN I signaling [118]. It is important to emphasize that in infectious diseases, the initial activation of STING is largely mediated by the cGAS-dependent antiviral pathway, whereas abnormal and persistent activation following cell death more often drives pathological inflammation. Such activation may result from multiple overlapping mechanisms, including mtDNA-induced cGAS activation as well as the aforementioned PRRs-independent modes of activation and regulation. Differentiating these contexts is crucial for disease classification and therapy [125].

**Figure 3:**
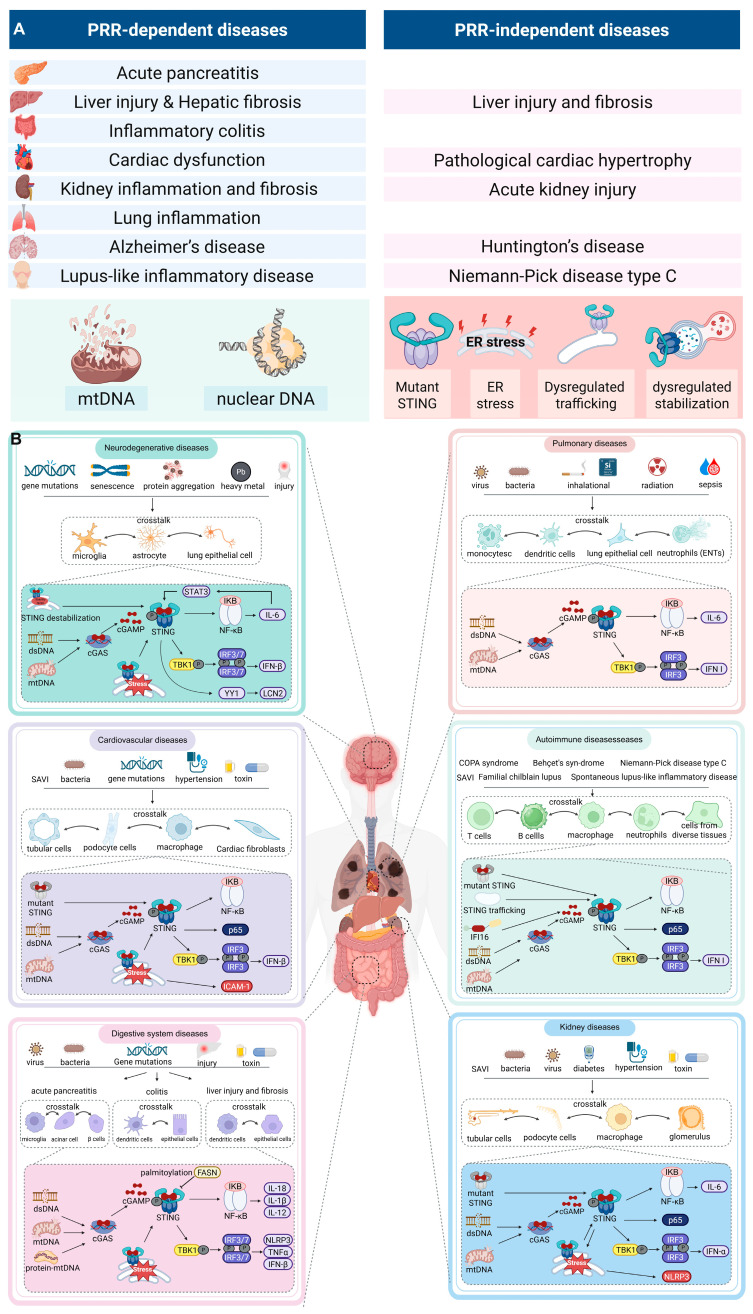
PRRs-dependent and independent mechanisms of STING activation across diseases. (**A**) Left panel: PRRs-dependent activation, in which cytosolic DNA derived from mitochondria (mtDNA), nuclei (nDNA), or microbes is sensed by cGAS to generate cGAMP, leading to STING activation. Representative diseases include myocardial infarction, systemic lupus erythematosus, acute kidney injury, and acute lung injury. Right panel: PRRs-independent activation, which occurs independently of cGAS through mechanisms such as STING gain-of-function mutations (SAVI), ER stress, defective ER–Golgi trafficking (COPA syndrome), impaired lysosomal degradation (Niemann–Pick disease type C), and cellular stress or death–associated signals. Representative diseases include SAVI, COPA syndrome, neurodegenerative disorders, and chronic autoinflammatory conditions. (**B**) STING is activated by genetic mutations, pathogens, toxins, and metabolic stress in both immune cells (macrophages, dendritic cells, T cells, B cells, neutrophils) and tissue-resident cells (epithelial cells, fibroblasts, podocytes, tubular cells). Crosstalk between these cell types amplifies STING-driven type I interferon and NF-κB signaling, promoting chronic inflammation, autoimmunity, and tissue injury.

The prevailing paradigm of STING activation in disease emphasizes PRRs dependence, yet new findings challenge this view by demonstrating widespread PRRs-independent STING activation. Figure 3 and Table 2 present a taxonomy of STING-associated diseases, distinguishing between PRRs-dependent and independent mechanisms.

### 4.1. Digestive System Diseases

Activation of the cGAS-STING pathway has been implicated in various inflammatory conditions of the digestive system. In acute pancreatitis (AP), STING deficiency attenuates NLRP3-mediated macrophage pyroptosis, reduces IRF7 expression, and disrupts the nuclear translocation of phosphorylated IRF3 (p-IRF3) [126]. As early as 2018, Zhao et al. have found that STING was significantly upregulated in pancreatic tissue and macrophages infiltrating the pancreas, with macrophages being the primary source of STING [127]. Adaptive immunity significantly contributes to AP pathogenesis, with T cells mediating neutrophil and monocyte infiltration [128]. T cell receptor (TCR) activation upregulates STING in T cells, initiating TBK1/STING/IRF3 phosphorylation and IFN I production. This IRF3-dependent mechanism impairs T cell proliferation and differentiation [129]. STING activation-mediated liver diseases have been reported. The crosstalk of STING among Hepatic stellate cells (HSCs), hepatocytes, and immune infiltrating cells (such as macrophages) drives the progression of hepatitis and liver fibrosis [130,131,132]. The buildup of STING in intestinal macrophages and monocytes promotes persistent inflammation, dysbiosis, and fibrosis in the gut [133]. In inflammatory bowel disease, STING accumulation drives chronic inflammation, dysbiosis, and fibrosis in the gut [133]. Dendritic cells detect gut microbial DNA [134,135], while damaged intestinal cells release nuclear/ mitochondrial DNA (mtDNA) that activates macrophage STING via exosomes [136] and dendritic cells (DCs) [137]. ER stress represents a major mechanism of STING activation, with the STING-IRF3 signaling axis mediating the crosstalk between ER stress and apoptosis [138,139].

### 4.2. Cardiovascular Diseases

During myocardial infarction, damaged cardiomyocytes release mtDNA/dsDNA, which activates the cGAS-STING pathway, subsequently inducing the production of interferons and cytokines [140,141]. In cardiovascular diseases, the release of mtDNA directly triggers cGAS-STING activation in vascular endothelial cells [142,143], while also recruiting immune cell infiltration [144] and promoting cell polarization [145], This process exacerbates vasculitis, atherosclerosis and cardiac dysfunction [146,147].

Moreover, STING activation in non-DNA damage contexts also critically contributes to cardiovascular disease progression. SAVI, a STING mutation-induced systemic autoinflammatory disease, encompasses cardiovascular diseases in early life [68]. Normally, STING is activated through cGAS; however, gain-of-function mutations disrupt normal immune development [59], impairing infection defense and driving pathological inflammation [97]. In STING-mutant patients, constitutive NF-κB activation in monocytes triggers early T cell activation, causing their aging and programmed cell death [148]. This leads to marked depletion of αβ T cells in blood and spleen, along with compensatory expansion of γδT cells [59], ultimately causing an inflammatory syndrome. ER stress-mediated non-canonical STING activation also drives various cardiac diseases, such as angiotensin II (Ang II) and aortic constriction (AC)-mediate cardiac inflammation and fibrosis [74], ischemia/reperfusion induced- myocardial injury [77] and hyperlipidemia induced-microvasculitis [78].

### 4.3. Kidney Diseases

The cGAS-STING pathway triggers damage to renal cells, contributing to the development of urological disorders. Renal mtDNA injury triggers STING activation, leading to parenchymal inflammation [149,150,151] and recruiting immune cells (e.g., neutrophils/M1 macrophages), which exacerbate disease progression [152]. Notably, STING activation exhibits sexual dimorphism due to three prime repair exonuclease 1 (TREX1)-dependent cytosolic DNA clearance [153]. In lupus nephritis-associated end-stage renal disease, the cGAS-IFI16-STING pathway triggers IRF3-mediated apolipoprotein L1 and interferon-β (IFN-β) expression via dsDNA [154]. ER stress initiates STING hyperactivation [155], establishing a feedforward loop wherein STING signaling potentiates ER stress through protein kinase R-like ER kinase (PERK) in kidney [156]. Additionally, dysregulated STING signaling promotes renal injury in SAVI [157]. Although some reports suggest that acyl-coenzyme A (CoA) synthetase long-chain family member 4 (ACSL4) can bind to STING to promote its activation under oxidative stress, cGAS appears to play a certain role in this process. It remains unclear whether, in the absence of cGAS, other molecules can compensate for its function in activating STING [158]. Further research is warranted to elucidate the roles of various factors in kidney-related diseases under physiological conditions or in the absence of PRRs such as cGAS. Understanding these mechanisms could pave the way for novel therapeutic strategies to mitigate renal injuries associated with STING overactivation.

### 4.4. Pulmonary Diseases

Studies have demonstrated that the cGAS-STING pathway plays a pivotal role in exacerbating pulmonary diseases, including silicosis [159,160,161], acute lung injury (ALI) [126,162,163,164], radiation-induced lung injury [165,166], and COVID-19 [167,168]. In silicosis patients, damaged lung tissues release nuclear dsDNA, which activates M1/M2 macrophages and DCs. Additionally, mtDNA within DCs can initiate STING pathway in these cells [160,161]. Acute respiratory distress syndrome (ARDS) is a sudden, widespread inflammatory injury to the lungs caused by factors including pneumonia, infections outside the lungs, trauma, blood transfusions, and burns. Emerging evidence demonstrates that infection-induced ALI is mediated through the neutrophil extracellular traps (NETs)-cGAS-STING axis, where NETs trigger STING activation in both neutrophils and pulmonary epithelial cells [164,169,170]; autophagy impairment in lung cells promotes mtDNA release, amplifying STING signaling [126,171]. Macrophage recruitment and activation critically contribute to this pathogenic cascade [126]. Although STING-mediated IFN I pathway is indispensable for antiviral innate immunity [172], excessive STING activation due to tissue inflammation and injury later exacerbates cytokine storms [167,168,173]. In aforementioned SAVI patients, ILD is also a prominent feature [15]. This review provides a detailed explanation of STING-mediated lung inflammation [174].

### 4.5. Neurodegenerative Diseases

Factors like oxidative stress, excitotoxicity, and neuroinflammation are key contributors to neurodegenerative diseases. In the central nervous system, microglia and astrocytes exhibit elevated expression of cGAS and STING, with the cGAS-STING signaling pathway activated in various neurodegenerative conditions [175]. In Alzheimer’s disease, toxic protein aggregates (e.g., amyloid β and p-tau) induce mitochondrial damage and DNA breaks, releasing dsDNA that activates cGAS-STING pathway, thereby promoting neuroinflammation and neurodegeneration [176,177,178,179]. Inhibiting cGAS-STING reduces inflammation and improves cognition, suggesting its therapeutic potential [176,180,181,182]. In Parkinson’s disease, neuroinflammation driven by neurons and glial cells contributes to dopaminergic neuron degeneration and dopamine reduction [183]. Damaged neurons release dsDNA and mtDNA, activating cGAS-STING and exacerbating parkinsonism [184,185,186]. This pathway is also implicated in other neurodegenerative disorders, including ischemic stroke [187], Huntington’s disease [188], and amyotrophic lateral sclerosis (ALS) [189]. Non-canonical (dsDNA-independent) STING activation pathways drive disease progression in neurodegenerative disorders. ER stress in neurons triggers microglial M1 polarization and Th1 cell conversion, fostering neuroinflammation [79].

### 4.6. Autoimmune Diseases

Although the aforementioned systems include some autoimmune diseases, it is necessary to discuss STING activation separately due to its critical role in autoimmune disorders. Dysfunction of host deoxyribonucleases (Dnases) leads to the abnormal accumulation of intracellular DNA, which hyperactivates the STING signaling pathway and contributes to autoimmune diseases. Normally, dsDNA is restricted to the nucleus and mitochondria, where it is transcribed into mRNA that is subsequently transported to the cytoplasm for protein synthesis, where it is transcribed into mRNA and transported to the cytoplasm for protein synthesis. However, various cellular stressors, such as replication errors, necrosis, apoptosis, and nuclear membrane rupture, can cause the release of DNA fragments into cytoplasm. These cytosolic DNA fragments are recognized by cGAS, initiating an immune response for clearance. Host Dnases, including TREX1, ribonuclease HII complex, SAM and HD domain containing protein 1 (SAMHD1), adenosine deaminase acting on RNA 1 (ADAR1), interferon induced with helicase C domain 1 (IFIH1), U7 small nuclear RNA associated protein (LSM11), and U7 small nuclear 1 (RNU7-1), are responsible for degrading this abnormally accumulated DNA, thereby preventing autoimmunity. Dysfunction in these nucleases disrupts cytoplasmic DNA metabolism, causing sustained activation of the cGAS-STING signaling pathway, leading to tissue inflammation and autoimmune conditions.

TREX1, located on the cytoplasmic face of the ER, functions as a 3′ to 5′ exonuclease, degrading cytosolic DNA to prevent persistent cGAS activation and autoimmunity [121,122,190,191,192]. Mutations or deletions in TREX1 result in the cytosolic DNA buildup [192], initiating autoimmune responses associated with conditions such as Aicardi-Goutières syndrome (AGS), systemic lupus erythematosus (SLE), familial chilblain lupus, retinal vasculopathy with cerebral leukodystrophy, and renal thrombotic microangiopathy [193]. TREX1-deficient (*Trex1*^−/−^) mice develop inflammatory conditions, including myocarditis and neuroinflammation, driven by elevated IFN I levels and increased ISGs expression [194]. Notably, the autoimmune symptoms in these mice can be reversed by deleting the STING or cGAS genes, which prevents type I IFNs (IFN I) production and reduces tissue inflammation [122,195,196].

Mutations in other DNases, such as DNASE1, DNASE1L3, and DNASE2, have also been linked to systemic autoimmunity. For instance, biallelic mutations in the DNASE2 gene, cause a loss of endonuclease activity, leading to high levels of type I IFNs and ISG expression in lymphocytes and monocytes. In mice, deletion of Dnase2 causes embryonic lethality due to anemia and excessive IFNs production, which can be reversed by deleting cGAS, STING, or IFNAR1. Additionally, mutations in ADAR1, IFIH1, LSM11, and RNU7-1 have been associated with autoimmune and autoinflammatory diseases such as AGS, SLE, and bilateral striatal necrosis (BSN), all characterized by aberrant type I IFNs production and systemic inflammation [197,198,199]. DNase dysfunction is closely tied to various autoimmune and inflammatory diseases in humans, though many underlying mechanisms remain poorly understood.

### 4.7. PRRs-Independent STING Activation Has Been Severely Underestimated

A critical but often overlooked issue is that STING activation persists in many disease models even when cGAS is genetically ablated [86]. This clearly indicates that STING can be triggered by a wide spectrum of PRRs-independent mechanisms, including gain-of-function mutations (e.g., SAVI) [68,98,200], ER stress [73,201], trafficking defects (COPA syndrome) [202,203,204], impaired lysosomal degradation (NPC disease) [86,205,206], and various forms of cellular stress and death [207]. However, much of the literature continues to emphasize DNA damage and cGAS-dependent activation. This bias is partly historical, as the cGAS-DNA-STING axis was the first and best-characterized pathway, and partly technical, since DNA leakage is easier to visualize and quantify using established assays compared to stress- or trafficking-related signals [27]. In addition, DNA damage provides an intuitive pathological link in ischemic, infectious, and autoimmune models, which makes it a more accessible focus for publication and recognition. As a result, the contribution of non-DNA triggers is consistently underestimated.

These findings strongly suggest that PRRs-independent mechanisms—such as STING gain-of-function mutations, ER stress, trafficking defects (e.g., COPA syndrome), impaired lysosomal degradation (e.g., Niemann–Pick type C), and cell death–associated stress responses—are critical but underappreciated drivers of STING signaling. We therefore urge that future studies systematically evaluate STING activation under cGAS-deficient conditions, in order to uncover the full spectrum of upstream triggers. By doing so, the field will avoid over-reliance on the DNA damage paradigm and better capture the clinically relevant contexts—particularly in chronic autoinflammatory and degenerative diseases—where STING itself functions as the central signaling hub and the most rational therapeutic target.

**Table 2:** PRRs-dependent and independent diseases associated with STING activation.

System Diseases	Activated Cells/Tissue	Trigger	Mechanism of STING Activation	PRRs/Non-PRRs
Digestive system diseases				
Severe acute pancreatitis	Macrophages	mtDNA	mtDNA―cGAS―STING―IRF3/IRF7―NLRP3―SAP [126].	cGAS
Acute pancreatitis	Macrophages	Cytosolic DNA of acinar cells	Cytosolic DNA/cell―free DNA―cGAS―STING―NF kB/IRF3―TNFα/IFNβ―AP [127].	cGAS
Acute pancreatitis	Pancreatic acinar cells	mtDNA of acinar cells	cGAS―STING―NF-κB―AP [208].	cGAS
Acute pancreatitis	Pancreatic β cells	Inflammatory mitochondria	Inflammatory mitochondria from macrophages―cGAS―STING activation [209].	cGAS
Acute liver injury	Macrophages	mtDNA	mtDNA―cGAS―STING―NLRP3―hepatocyte pyroptosis [131].	cGAS
Hepatic inflammation and fibrosis	Macrophages	cGAMP	cGAMP―STING―JNK1/NFκB―hepatocyte and HSC inflammatory and fibrosis [132].	cGAMP
Inflammatory colitis	Monocytes	cGAS/CDNs	cGAS/microbiota―STING―MyD88―IFNβ/IL1β/IL18―inflammatory colitis [135].	cGAS
Insulin resistance and inflammatory colitis	Hepatocytes, adipocytes and macrophages	DNA from mEVs	mEVs―cGAS-STING―insulin resistance and inflammatory colitis [136,210].	cGAS
Colitis	Dendritic cells	Protein-mtDNA complex from damaged colonic epithelial cells	protein-mtDNA complex―STING―IRF3/NF-κB―IL-12 (T helper cell)―chronic colitis [137].	protein-mtDNA complex (DAMPs)
Liver fibrosis	Hepatocytes	TNF-α	TNF-α―STING―IRF3/WDR5/DOT1L―NLRP3―GSDMD [130].	cGAS
Alcoholic liver disease	Hepatocytes	ER stress	ER stress―STING―IRF3―hepatocyte apoptosis [138].	non-PRRs
Liver injury and fibrosis	Hepatocytes	ER stress	ER stress―STING―IRF3―hepatocyte apoptosis [139]	non-PRRs
Liver metaflammation	Macrophages	STING trafficking	SCAP―STING―TBK1―NF-κB―metaflammation [87]	non-PRRs
Liver injury	Macrophages	Malonyl-CoA/FASN	Malonyl-CoA inhibits STING palmitoylation to alleviate sepsis-induced liver injury [211].	non-PRRs
Cardiovascular diseases				
Vascular diseases induced by obesity	Aortic endothelial cells	mtDNA	mtDNA―cGAS―STING―IRF3―ICAM-1―endothelial activation/inflammation [142].	cGAS
Atherogenesis	Macrophages	mtDNA	mtDNA―cGAS―STING―TBK1―NF-κB―atherogenesis [146].	cGAS
Cardiac dysfunction	Ventricular myocytes & Macrophages	mtDNA	iNOS―mtDNA―cGAS―STING―IRF3―cardiac dysfunction [145].	cGAS
Vascular endothelial dysfunction	Artery endothelial cells	Self-mtDNA	cGAS―STING―PERK―IRF3/NF-κB―vascular endothelial dysfunction and atherosclerosis [212].	cGAS
SAVI associated myocarditis	Monocytes & endothelial cells	Mutant STING	Mutant STING―NF-κB/IFN-β―inflammation [15,148].	non-PRRs
Pathological cardiac hypertrophy	Cardiac fibroblasts	ER stress	AC or Ang II―ER stress―STING-TBK1-RIF3/ NF-κB heart inflammation and fibrosis [74].	non-PRRs
Myocardial ischemia/reperfusion	Cardiomyocyte	ER stress	ER stress―STING-IRF3―Rubicon―autophagic flux dysfunction [72]	non-PRRs
Diabetic retinopathy	Retinal endothelial	ER stress	Hyperlipidemia―IRE1α―XBP1―ER stress―STING―TBK1―NF-κB―pro-inflammatory [78].	non-PRRs
Kidney diseases				
Acute kidney injury	Tubular cells	mtDNA	mtDNA―cGAS―STING―TBK1―p65―neutrophil infiltration and tissue inflammation [149].	cGAS
Glomerular diseases	Podocyte cells	mtDNA	cGAS―STING―TBK1―IRF3/IFN α―glomerular diseases [213].	cGAS
Kidney inflammation and fibrosis	Macrophages	dsDNA	dsDNA―cGAS―STING―TBK1/IRF3―p65 axis suppresses kidney inflammation and fibrosis [152].	cGAS
Acute kidney injury	Tubular cells	ER stress crosstalk	STING―ER stress―mtROS―NLRP3 inflammasome―acute kidney injury [155].	non-PRRs
SAVI-associated glomerulosclerosis	Glomerulus	Mutant STING	Mutant STING induces glomerulosclerosis [157].	non-PRRs
Pulmonary diseases				
Lung inflammation	Monocytes, macrophages & dendritic cells	dsDNA	SiO_2_―dsDNA―cGAS―STING―IFN I pathway drives inflammation [161].	cGAS
Silica particles	Macrophages & fibroblasts	dsDNA	SiO_2_―dsDNA―cGAS―STING―IRF3/NF-κB―macrophage polarization―lung fibroblast proliferation [160].	cGAS
Lung injury	Lung epithelial cells & neutrophils	dsDNA from NETs	NETs―cGAS―STING pathway promotes inflammatory injury [164,169,170].	cGAS
Acute lung injury	Lung epithelial cells	mtDNA	Mitophagy―mtDNA―cGAS―STING―IFN I/IL-6―ALI [162,171].	cGAS
Neurodegenerative diseases				
Alzheimer’s disease	Microglia	Aβ, tau and APOE (Apolipoprotein E) ε4-DNA	Aβ, tau and APOE (Apolipoprotein E) ε4-DNA―cGAS―STING―TBK1―IRF3/NF-κB―neuroinflammation and phospho-tau levels [214,215].	cGAS
Alzheimer’s disease	Astrocytes	DNA	IL-6―STAT3 regulates cGAS―STING―neuroinflammation [216].	cGAS
Parkinson’s disease	Astrocytes	mtDNA	cGAS―STING―YY1―LCN2―neurodegeneration [185].	cGAS
Parkinson’s disease	Microglia	mtDNA	cGAS―STING―IRF7―neuroinflammation in Parkinson’s disease [217].	cGAS
Huntington’s disease	T cells, B cells, Dendritic cells & MEFs	STING destabilization	TOLLIP―STING―IRF3―neuroinflammation [58].	non-PRRs
Traumatic brain injury	Neurons	ER stress induced PERK	PERK―ER stress―STING―TBK1―IRF3―IFN-β―white matter injury [79].	non-PRRs
Autoimmune diseases				
Spontaneous lupus-like inflammatory disease	Systemic tissue	dsDNA induced by mutant TREX1	Mutant TREX1-dsDNA―cGAS―STING―spontaneous lupus-like inflammatory [190].	cGAS
Familial chilblain lupus	Dendritic cells	Mutant STING	Mutant STING―IFN I―familial chilblain lupus [63,218].	non-PRRs
Autoimmune and autoinflammatory diseases	Murine embryonic fibroblasts	STIM1 deletion or mutation	STIM1 deletion or mutation―STING―IFN―inflammation [77]	non-PRRs
Niemann-Pick disease type C	Various cells (e.g., monocytes, macrophages, neurons, fibroblasts)	STING trafficking	NPC1 deficiency―SREBP2 activation―STING―inflammation [86]	non-PRRs
COPA syndrome	Various cells (e.g., dendritic cells, macrophages, neurons, fibroblasts)	STING trafficking	COPA mutant―STING stay and palmitoylation―TBKI―inflammation [90,91]	non-PRRs
Behçet’s syndrome	Endothelial cells, neutrophils	dsDNA	TNF-α upregulated IFI16―STING―TBK1―apoptosis of intestinal epithelial cells [35].	IFI16

## 5. Current Therapeutics to Target STING Signaling

**Figure 4:**
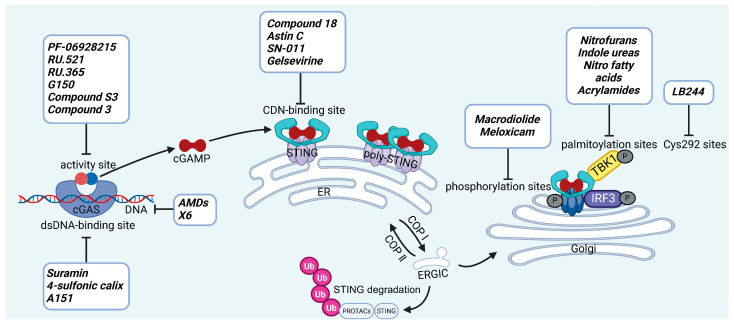
The representative inhibitors in the STING signaling pathway and their inhibition sites. The inhibition of the STING signaling pathway can be categorized based on specific mechanisms. (1) Inhibition of cGAS enzymatic activity: Blocking the enzyme function of cGAS to prevent the synthesis of cGAMP in response to cytosolic DNA; (2) Prevention of DNA-induced cGAS activation: Targeting the DNA recognition process, preventing cGAS from detecting and binding to foreign or damaged DNA; (3) Blocking cGAMP binding to STING: Inhibiting the interaction between cGAMP and STING, thus preventing the activation of STING; (4) Disruption of downstream STING signaling: Inhibiting key post-translational modifications such as STING phosphorylation, palmitoylation, and the modification of Cys292, which are critical for the propagation of the signaling cascade. (5) Induction of STING ubiquitin-proteasome degradation: Covalent dual binding of small molecules to both STING and E3 ubiquitin ligase promotes the ubiquitination of STING from the ER to the Golgi apparatus, thereby inhibiting the process of STING reaching the Golgi. (Single-headed arrow indicates direction of flow, and a bar-headed arrow indicates inhibition.).

cGAS is a key PRRs that activates STING by catalyzing the cyclization of adenosine triphosphate (ATP) and guanosine triphosphate (GTP) into CDNs. In this section, we focus on the cGAS-STING signaling pathway and its therapeutic potential. Accumulating evidence suggests that targeting STING signaling in specific tissues and cell types offers promising strategies for the treatment of microbial infections and autoimmune diseases. A variety of small molecules and therapeutic approaches have been developed to inhibit STING signaling, which can be classified based on their mechanisms of action. These include: (1) inhibition of cGAS enzymatic activity, (2) prevention of DNA-induced cGAS activation, (3) blockade of cGAMP binding to STING, and (4) disruption of downstream STING signaling (Figure 4). Collectively, targeting the STING pathway via these inhibitors represents a novel and effective therapeutic approach for diseases associated with aberrant STING activation. A summary of these inhibitor categories is provided in Figure 4 and Table 3.

### 5.1. Inhibiting cGAS Enzymatic Activity

**PF-06928215.** PF-06928215 functions as an inhibitor targeting the catalytic site of cGAS, was discovered by researchers at Hall et al. It acts as a potent competitive inhibitor, vying with ATP and GTP for binding to the catalytic site of cGAS [219]. Wang et al. employed PF-06928215 to mitigate PM2.5 (particulate matter ≤2.5 μm)-induced cellular senescence in lung tissue by suppressing inflammation mediated through the cGAS-STING-NF-κB signaling pathway [220].

**RU.521 and RU.365** are small-molecule compounds that effectively bind to the catalytic site of cGAS. RU.365 reduces cGAS affinity for GTP and ATP but acts as a non-competitive inhibitor, whereas RU.521 shows stronger cGAS targeting, lower effective concentration, and greater competition with ATP [221]. Shao et al. employed RU.521 to promote neurological recovery and attenuate neuroinflammation by regulating microglial polarization through the cGAS-STING pathway during the early phase of brain injury post-subarachnoid hemorrhage [222].

**G150.** Lama et al. have screened G140 and G150, they demonstrate greater potency compared to RU.521 [223]. The key interactions involve the tricyclic core of G150, the guanidine group of Arg376, and the aromatic ring of Tyr436 in cGAS [224]. The compound exhibits specificity for human STING and shows robust therapeutic effects in humanized mouse models [225].

**Compound S3.** Zhao et al. identified compound **S3**, a cGAS inhibitor with potency comparable to RU.521. Structural analysis showed that **S3** forms hydrogen bonds with R376, N482, and S380 (water-mediated), π–π stacking with Y436, cation–π interactions with R376, and hydrophobic contacts via its 4,5,6,7-tetrahydrobenzo[b]thiophene core with H437, F488, L490, and L495 [226].

**Compound 3.** Song et al. showed that compound **3** forming a covalent bond at Cys419 and inhibiting enzymatic activity by blocking substrate access to the catalytic site. Compound **3** also alleviated DSS-induced colitis in mice, though its activity against human cGAS was weaker [227].

**CU-32 and CU-76.** In 2019, Hang et al. identified a small-molecule inhibitor of cGAS that inhibits its activity by disrupting cGAS dimerization. It was shown to decrease IFN-β production in murine macrophages [228].

**Compounds 30d-S.** Zhang et al. synthesized a novel cGAS inhibitor in 2024. In in vivo experiments, they demonstrated that it inhibits LPS (lipopolysaccharide)-induced ALI by suppressing the cGAS-STING pathway [229].

### 5.2. cGAS-dsDNA Binding Inhibitors

**AMDs.** Antimalarial drugs (AMDs) have been shown to impede the cGAS-STING pathway [230]. A key feature of AMDs is their interaction with nucleic acids, particularly dsDNA, suggesting they may inhibit cGAS by blocking DNA binding. However, the lack of direct correlation between AMD-DNA binding and cGAS inhibition implies a more complex mechanism [231]. Tonduti et al. have mentioned in a review that AMDs could be a strategy to treat Aicardi-Goutières syndrome [232]. But AMDs is rarely used to suppress cGAS because we have better options.

**X6.** An et al. have reported that a series of AMDs block the cGAS-dsDNA interaction. Based on AMDs they have developed a second-generation compound, X6, demonstrated stronger activity and provided greater amelioration of autoimmune myocarditis in vivo [233].

**Suramin.** Suramin probably inhibits cGAS activity by attaching to the dsDNA-binding site, thereby preventing the formation of the cGAS-dsDNA complex [234]. As with AMDs, Suramin is rarely used clinically as a cGAS inhibitor and remains restricted to in vitro experimental settings.

**4-sulfonic calix**[6]. Green et al. discovered that 4-sulfonic calix[6] can suppress the cGAS-STING pathway by targeting both the dsDNA-binding site on cGAS and 2′,3′-cGAMP. Notably, it alleviated AIM2-dependent inflammatory diseases in vivo [235].

**Suppressive oligodeoxynucleotides.** Suppressive oligodeoxynucleotides like A151 function as competitive inhibitors of cGAS by binding to its dsDNA-binding domain. They have been shown to suppress IFN I responses in TREX1-deficient cells. A151 consists of four repeats of the TTAGGG motif (5′-ttagg gttagg gttagg gttagg g-3′), which facilitates its inhibitory activity [50].

**XQ-2B.** XQ-2B is a macrocyclic peptide that directly binds to cGAS, thereby disrupting its interaction with double-stranded DNA (dsDNA). This compound was first identified in 2023 by Junmin Quan and colleagues as a selective cGAS inhibitor [236]. Their study showed that XQ-2B suppresses STING-mediated inflammation in Trex1 deficiency and reduces IFN-I production during HSV-1 infection in vivo.

### 5.3. Other cGAS Inhibitors

Through a survey of global databases, we identified several cGAS inhibitors that have advanced into clinical trials; however, neither detailed clinical trial data nor molecular structures have been publicly disclosed. VENT-03 is currently undergoing a phase I clinical trial in the Netherlands for the indication of SLE [237], IMSB-301 is currently undergoing Phase I clinical trials for SLE and cutaneous lupus erythematosus [238]. However, these compounds remain limited to early-phase studies.

### 5.4. STING Antagonists Targeting the CDN-Binding Site

**Tetrahydroisoquinolines.** Siu et al. capitalized on the symmetrical attributes of the CDN-binding domain to devise small-molecule antagonists, leading to the development of tetrahydroisoquinolines, especially Compound **18**. However, they are still at the in vitro [239].

**Astin C.** Li et al. discovered that astin C exhibited a binding affinity to STING-CTD-H232 comparable to that of CDNs. Specifically targeting STING, astin C competes with CDN for binding to the C-terminal activation site of STING. It alleviated autoimmune inflammation in mice [240].

**SN-011.** Hong et al. identified SN-011 that binds this site with higher affinity than 2′3′-cGAMP, stabilizes STING in an inactive state, and selectively suppresses STING-driven interferon and inflammatory responses without affecting other innate immune pathways in vitro [241].

**Gelsevirine.** Chen et al. reported that GS, an alkaloid from Gelsemium elegans, competitively binds STING, inhibits 2′3′-cGAMP-induced dimerization, and promotes K48-linked ubiquitination and proteasomal degradation, potentially via TRIM21. It alleviated inflammation in septic mice [242].

**T0901317.** Zhang’s team recently revealed T0901317, an LXR agonist that regulates lipid metabolism and specifically blocks 2′3′-cGAMP-mediated STING signaling. They further showed that SMPDL3A selectively cleaves 2′3′-cGAMP, highlighting how cGAMP metabolism regulates STING activation and providing new inhibitory targets [243].

**Anhydrotuberosin.** Lei’s team discovered a new small-molecule inhibitor that directly binds to the C-terminal of STING. This is a competitive inhibitor with a dose-dependent effect. This molecule inhibits SAVI-related STING activation, reducing IFN-I and pro-inflammatory cytokines in *Trex1*^−/−^ mice, and also alleviates DSS-induced colitis [244].

### 5.5. Targeting STING Phosphorylation Sites

**Macrodiolide.** The compounds LH531 and LH519 were able to inhibit STING phosphorylation during early time points, with LH531 showing sustained suppression of STING phosphorylation at later stages. These compounds may directly phosphorylate STING at Ser366, but both LH531 and LH519—especially LH531—also increase TBK1 phosphorylation independently of STING. It demonstrated strong inhibitory effects on STING in human BMDMs and in murine colitis models. [245].

**Meloxicam.** Yu et al. demonstrated that Meloxicam (MXC) significantly downregulates the expression of ISGs in TREX1-deficient cells. Moreover, MXC was shown to enhance survival in the *Trex1^−/−^* mouse model of Aicardi-Goutières syndrome. However, they did not reveal the specific mechanism by which MXC inhibits STING phosphorylation. Based on the p-STING antibodies used in their study, we inferred that MXC inhibits phosphorylation at Ser365 of mouse STING and at Ser366 of human STING [246]. Notably, although this drug has already been approved for the treatment of rheumatoid arthritis, it is not clinically used as a STING inhibitor. However, its newly identified action on STING may further explain its analgesic and anti-inflammatory effects.

**4-OI.** Ren et al. discovered that 4-OI alkylates cysteine residues 65, 71, 88, and 147 of STING, thereby inhibiting phosphorylation of STING, as well as, inhibiting the production of inflammatory factors in sepsis mouse models [247].

### 5.6. Targeting STING Palmitoylation Sites

Protein translocation across cellular membranes is frequently associated with lipid-mediated post-translational modifications, among which palmitoylation is a well-recognized example [248].

**Nitrofurans.** Haag et al. showed that a group of nitrofuran derivatives bind selectively and irreversibly to Cys91 of STING, without affecting the neighboring Cys88. Their studies indicated that the nitro group and the furan ring are crucial for the compounds’ activity, as exemplified by C-176, C-178, C-170, and C-171 which ameliorated inflammation in murine models of autoimmune disease [249].

**Indole ureas.** Compounds containing indole ureas have been demonstrated to form a covalent bond with Cys91. H151 could reduce IFN I responses in a mouse model of ischemia–reperfusion, blocking TBK1 phosphorylation and preventing the palmitoylation of STING at Cys91 [250].

**Nitro fatty acids.** Hansen et al. have shown that nitrooleic acid alkylated both Cys88 and Cys91 and alkylate of His16 to inhibit transduction of STIGN downstream signaling pathways in vitro [251].

**Acrylamides.** Vinogradova et al. discovered that acrylamide compounds BPK-21 and BPK-25 reacted with and formed adducts at Cys91 of STING [252].

**4-OI.** Yi et al. discovered a small-molecule inhibitor that targets palmitoylation of Cys91 on STING. This compound is an itaconate derivative, which reduces IFN I expression during HSV-1 infection, as well as inflammatory responses in *Trex1*^−/−^ Raw264.7 cells [253].

**NO2-cLA.** Hansen discovered that the inhibitor, nitro-conjugated linoleic acid (NO2-cLA), binds to STING Cys88 and Cys91 to inhibit palmitoylation. Although its effect has not been demonstrated in animal models, it successfully inhibits IFN I in SAVI-derived fibroblasts [251].

### 5.7. Targeting STING Cys292 Sites

**LB244.** Barasa et al. reported that BB-Cl-amidine suppresses STING signaling but has low proteome-wide selectivity. They developed LB244 by replacing the chloroacetamidine warhead with nitrofuran, achieving greater potency and selectivity, and validated it in vitro. Notably, LB244 inhibits STING via modification of Cys292, representing the first inhibitor to target this site [254].

### 5.8. Targeting STING Degradation

**Degrader-1.** Proteolysis-targeting chimeras (PROTACs) are a new technology that selectively degrade target proteins using the ubiquitin-proteasome system [255]. Recent studies have indicated that promoting the degradation of STING is regulated through the ubiquitin-proteasome system. The compound covalently links STING with an E3 ubiquitin ligase to form a ternary complex, which is then degraded by the proteasome to inhibit the activation of downstream STING signaling pathways in vitro [256].

**SP23.** Chen et al. reported a PROTAC that connects E3 ligase and STING to promote STING’s proteasomal degradation, which was validated in a mouse model of nephritis [257].

**UNC9036.** Liu’s team developed a compound that promotes proteasomal degradation of STING by forming STING-based PROTACs, thereby inhibiting STING activation in vitro [258].

### 5.9. Attenuating STING Trafficking

**4-PBA.** It is an FDA-approved adjunct therapy for pediatric urea-cycle disorders—dampens IRF3 activation by attenuating STING COPII-mediated trafficking, by competing with p24 cargo receptors for COPII binding. Motivated by the structural similarity between phenylpropanoid-derived phenolic acids and 4-PBA (“4-PBA-like” natural hydrocinnamic acids), Nan et al. screened related compounds and identified five that suppress the IFN-β response by modulating STING trafficking; among them, 4-phenylcinnamic acid exhibited the strongest inhibitory effect.

### 5.10. Clinical Status and Translational Challenges

Although numerous inhibitors have shown preclinical efficacy, translation into clinical practice remains limited. The clinical landscape of cGAS-STING inhibitors is still at an early stage. A few clinically approved drugs, such as meloxicam, suramin, nitrofurantoin, and diamidines (e.g., pentamidine), have been retrospectively recognized to modulate STING signaling, although their inhibitory effects have not yet been translated into approved indications. Only a limited number of agents have advanced to clinical trials, including the endogenous-derived nitro fatty acid CXA-10 (NCT04053543, NCT04125745, NCT03449524), which has entered phase II studies for inflammatory and renal diseases, and VENT-03 (EUCT2023-507504-31-00) and IMSB-301 (ISRCTN90049550), first-in-class human STING modulators currently in phase I trials for autoimmune disorders. In contrast, most reported compounds, including natural products (astin C, Gelsevirine, anhydrotuberosin, macrodiolides, 4-OI, NO_2_-cLA) and synthetic molecules (LB244, Degrader-1, indole ureas, acrylamides), remain in the preclinical stage, where efficacy has been demonstrated in vitro and in animal models but comprehensive pharmacokinetic, safety, and translational data are lacking.

Despite these advances, several critical challenges hinder clinical translation. First, species-specific differences in STING structure limit the predictive value of murine studies; for example, the widely used inhibitor H-151 is potent in mice but much less effective in human cells, underscoring the need for inhibitors optimized for human STING and even population-specific variants. Second, selectivity and off-target risks remain a concern, as broad suppression of STING signaling could impair innate immunity and increase susceptibility to infection. Third, toxicity and safety require careful evaluation, since candidate molecules must demonstrate low toxicity, metabolic stability, and predictable pharmacokinetics while maintaining sufficient potency.

To address these limitations, emerging approaches are focusing on non-PRRs-mediated STING modulation, which bypasses the complexity of diverse upstream ligands and targets post-translational modifications or downstream signaling nodes. In addition, nanoparticle-based delivery systems and combination therapies may enhance stability, tissue specificity, and therapeutic efficacy, offering opportunities for more precise and safer modulation of STING activity. Taken together, these findings indicate that although the development of cGAS-STING inhibitors remains in its infancy, ongoing advances in drug design, delivery technologies, and translational strategies are steadily paving the way toward clinically viable therapies (Table 4).

## 6. Conclusions and Perspectives

The STING signaling pathway functions as a rapid-response mechanism to pathogenic insults and endogenous danger signals, playing a vital role in host defense and disease progression. While STING activation effectively combats infections, its dysregulation may lead to excessive inflammation and tissue injury. This review primarily outlines the mechanisms of STING activation and its roles in inflammatory diseases, and further summarizes current inhibitors targeting the STING pathway along with their respective mechanisms of action. In recent years, considerable progress has been made in elucidating the structural basis and regulatory dynamics of cGAS and STING, as well as the physiological significance of PRRs-dependent cGAS–STING signaling in various disease contexts. However, mounting evidence suggests that STING acts not only as a downstream adaptor in cytosolic DNA sensing but also as a central signaling hub integrating multiple cellular cues—such as genetic mutations and ER stress—that may trigger PRRs-independent activation. Such aberrant activation can initiate cytokine storms and pathological inflammation, contributing to the onset and progression of numerous autoimmune and inflammatory diseases. Therefore, pharmacologically targeting STING presents distinct therapeutic advantages compared to cGAS inhibition.

The pivotal role of the cGAS-STING pathway in inflammation is indisputable, yet its clinical translation remains challenging. Reliable biomarkers of pathway activation are still lacking, reflecting the heterogeneity of upstream triggers—ranging from microbial DNA and self-DNA leakage to ER stress and trafficking defects—and the diversity of downstream outputs, including IFN I, NF-κB, and inflammasomes. These signatures differ across diseases, are highly dynamic and context-dependent, and are further complicated by assay variability and limited specificity, making patient monitoring and stratification difficult.

Therapeutic targeting of the cGAS-STING axis faces similar obstacles. Disease heterogeneity means that STING is a dominant pathogenic driver in some settings (e.g., SAVI or TREX1 deficiency) but only one of many contributors in others, limiting the efficacy of single-pathway inhibition. Inter-individual variability, driven by genetic and immunological factors, reinforces the need for predictive biomarkers and precision medicine approaches. Importantly, suppressing STING carries the risk of compromising host defense against infections and tumors, while issues of drug specificity, durability, and toxicological evaluation remain unresolved. Given the redundancy of innate immune signaling, cGAS-STING blockade alone may not be sufficient. Instead, treatment strategies should be tailored to disease type and patient characteristics, potentially combining inhibitors that act at different nodes of the cGAS-STING pathway or across complementary inflammatory pathways. Such context-specific and multifaceted approaches, coupled with rigorous biomarker development and long-term safety assessment, will be essential to achieve effective and safe translation of cGAS-STING-targeted therapies into clinical practice.

This review highlights several small-molecule inhibitors of cGAS and STING, emphasizing their mechanisms of action. Nonetheless, these agents require further optimization in key aspects, including improving target selectivity and potency, enhancing bioavailability and pharmacokinetics, minimizing toxicity, formulating for better patient compliance, and exploring synergistic combinations with existing therapies to enhance efficacy and reduce resistance. PRRs-independent STING activation remains a complex yet pivotal area of research, involving ER stress, protein trafficking and stabilization, and gene mutations. Continued investigation into the structural and mechanistic features of STING, its crosstalk with other signaling pathways, and organelle-specific regulation will deepen our understanding of its role in immune homeostasis. Ultimately, this knowledge will facilitate the development of novel therapeutic strategies for treating STING-driven inflammatory and autoimmune disorders.

## Figures and Tables

**Table 3:** Clinical development status of cGAS and STING inhibitors.

Drug (Code Name)	Target	Species	Clinical Phase	Indication	Trial Identifier
VENT-03	cGAS	Human	Phase 1	Lupus	EUCT2023-507504-31-00
IMSB-301	cGAS	Human	Phase 1	Lupus	ISRCTN90049550
CXA-10	STING (palmitoylation)	Human	Phase 2	Pulmonary Arterial Hypertension	NCT04053543 NCT04125745 NCT03449524

**Table 4:** Targeting the cGAS-STING pathway: preclinical inhibitors and therapeutic applications.

Reagent	Type	Effects	Structure	Therapeutics	References
cGAS catalytic site inhibitors					
PF-06928215	Substrate-competitive inhibitor	Inhibiting catalytic site of cGAS (human)	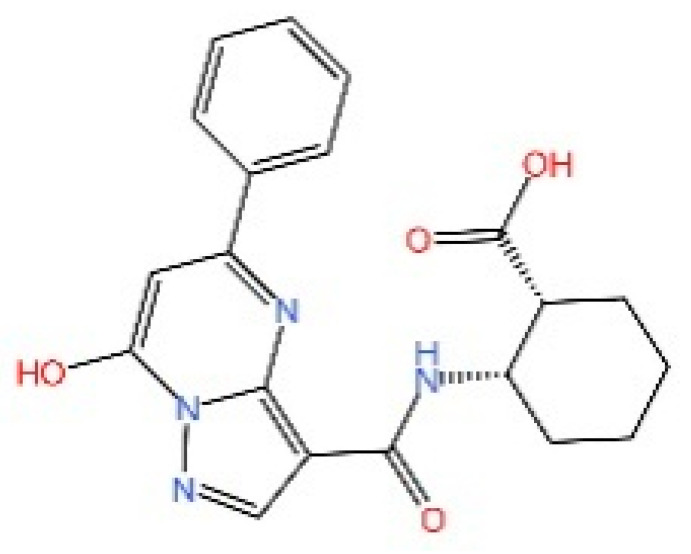	Ameliorating PM2.5-induced cellular senescence in the lung	[219,220,226]
RU.521	Substrate-competitive inhibitor	Inhibiting catalytic site of cGAS (mouse)	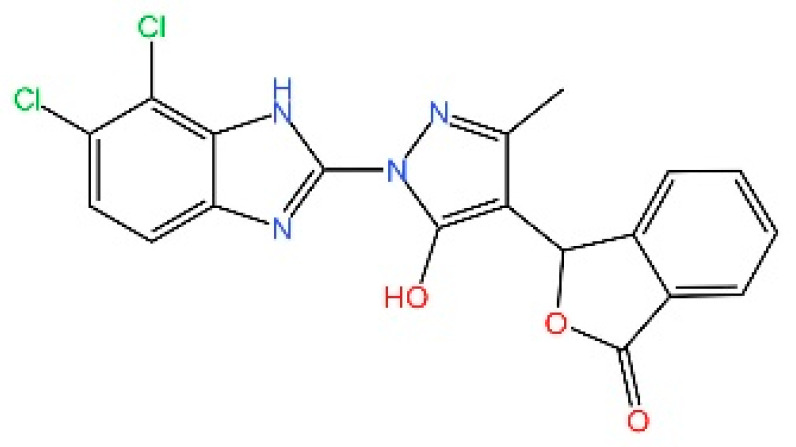	Mitigating subarachnoid hemorrhage-induced brain injury	[221,222]
G150	Non-covalent inhibitor	Inhibiting catalytic site of cGAS (human)	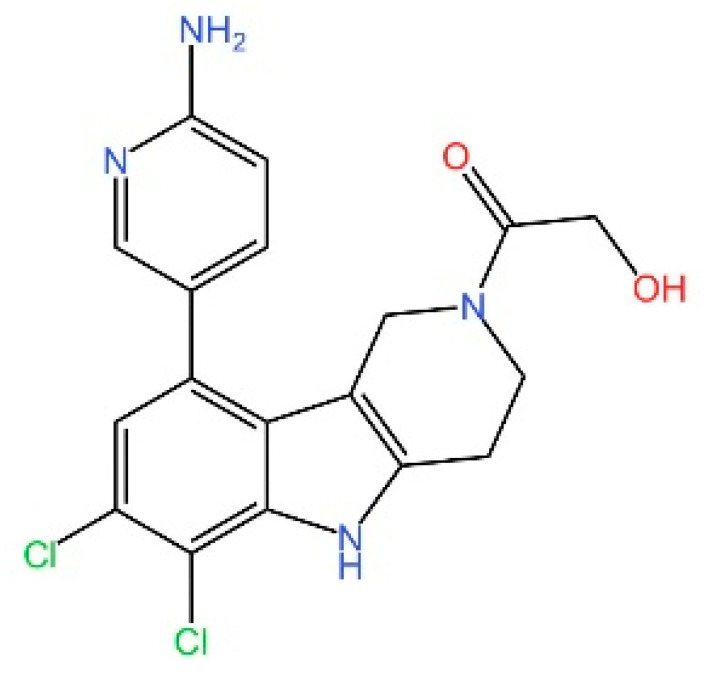	Alleviating polystyrene microplastics induce pulmonary fibrosis	[223,259]
Compound **S3**	Non-covalent inhibitor	Inhibiting catalytic site of cGAS (human)	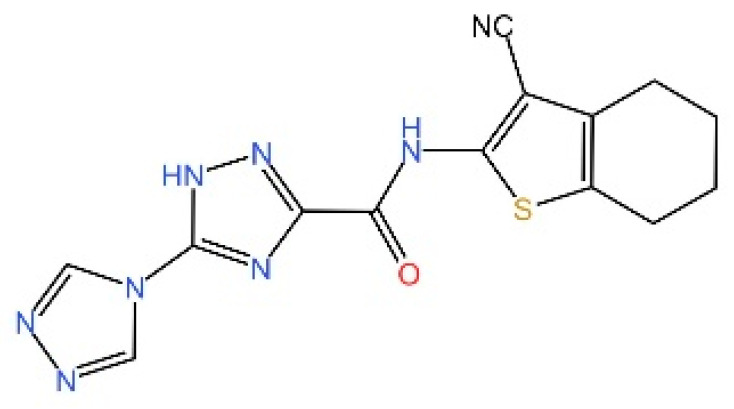	Treatment of Acute Lung Injury	[226,229]
Compound **3**	Covalently modified inhibitor	Inhibiting catalytic site of cGAS (mouse)	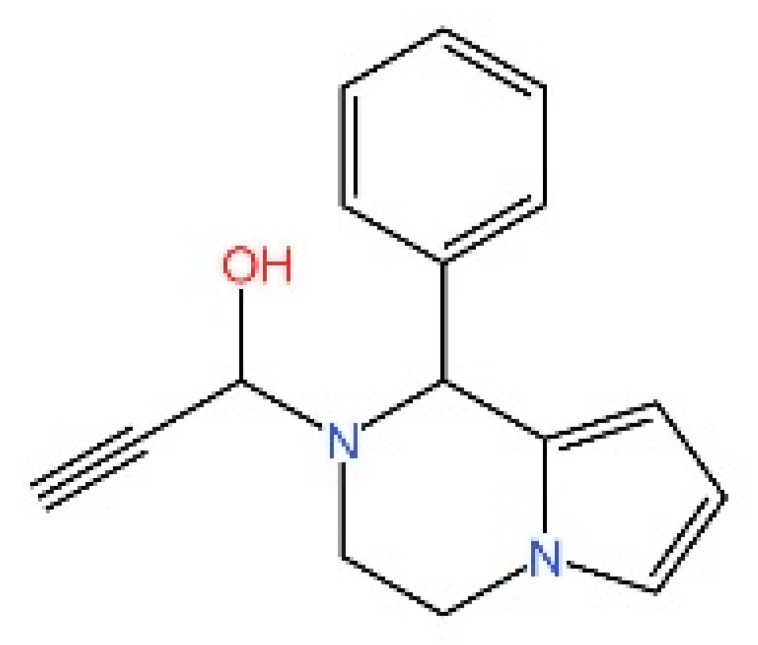	Alleviating DSS-induced colitis	[227]
CU-32 & CU-76	Substrate-no-competitive inhibitor	Inhibiting catalytic site of cGAS (human)	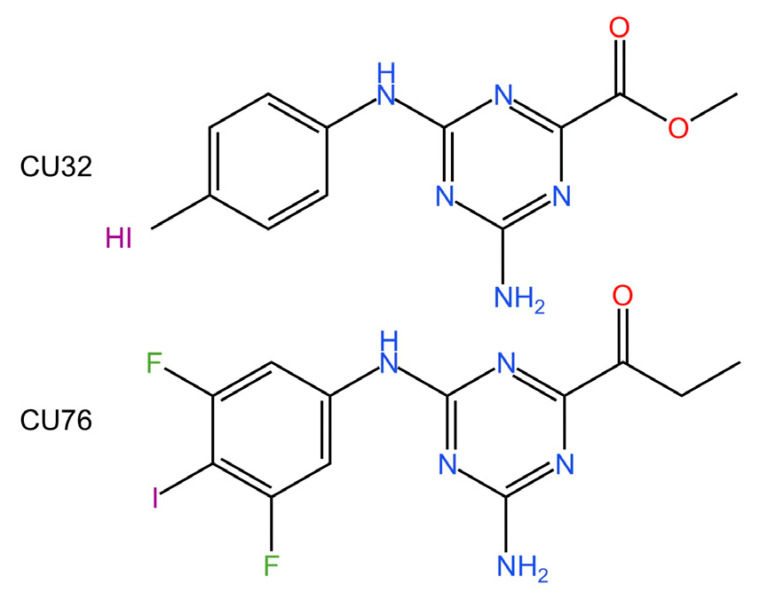	Reducing IFN-β production in macrophages	[228]
Compounds **30d-S**	Substrate-competitive inhibitor	Inhibiting catalytic site of cGAS (human)	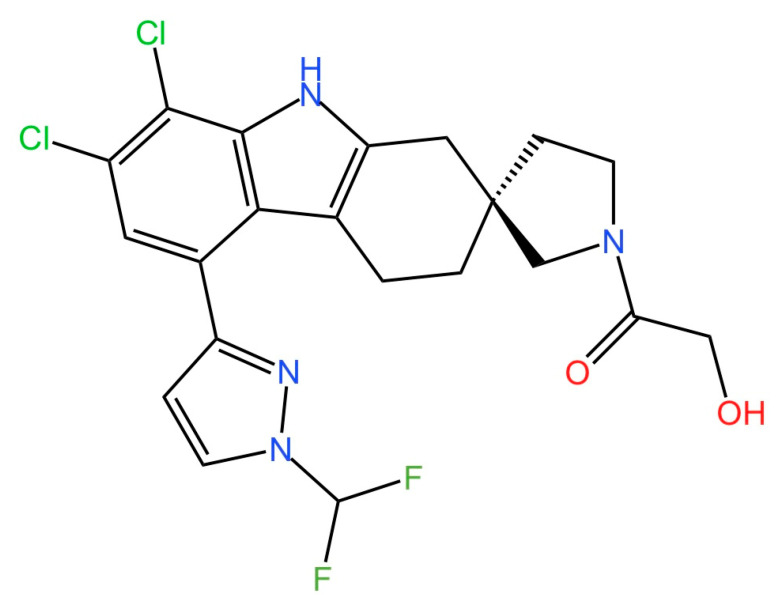	Alleviating LPS-Induced Acute Lung Injury (ALI) In Vivo.	[229]
cGAS-dsDNA banding inhibitors					
Quinacrine	Binding to dsDNA	Inhibiting cGAS-DNA interaction (human & mouse)	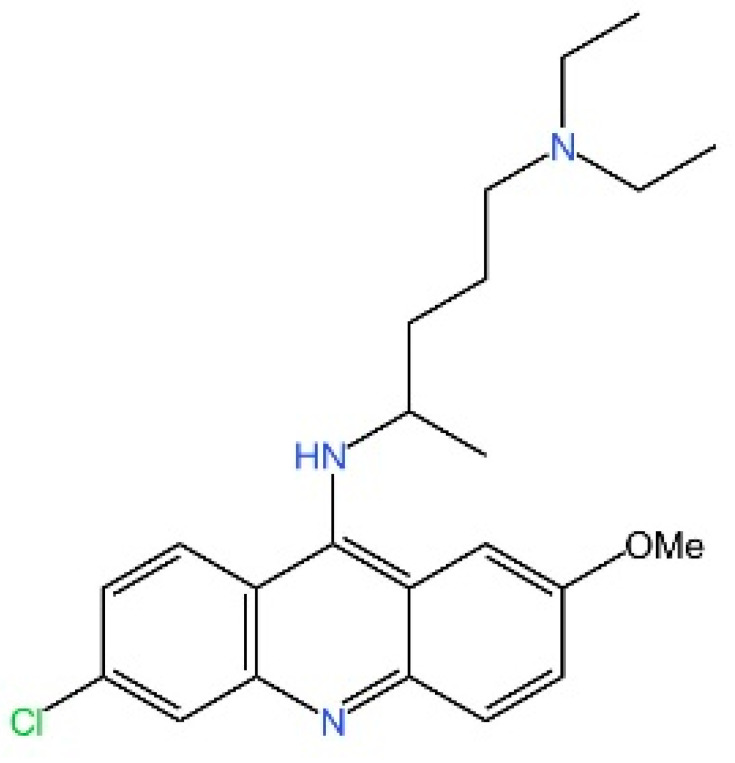	Reducing IFN-β production in vitro	[231,260]
X6	Binding to dsDNA	Inhibiting cGAS-DNA interaction (mouse)	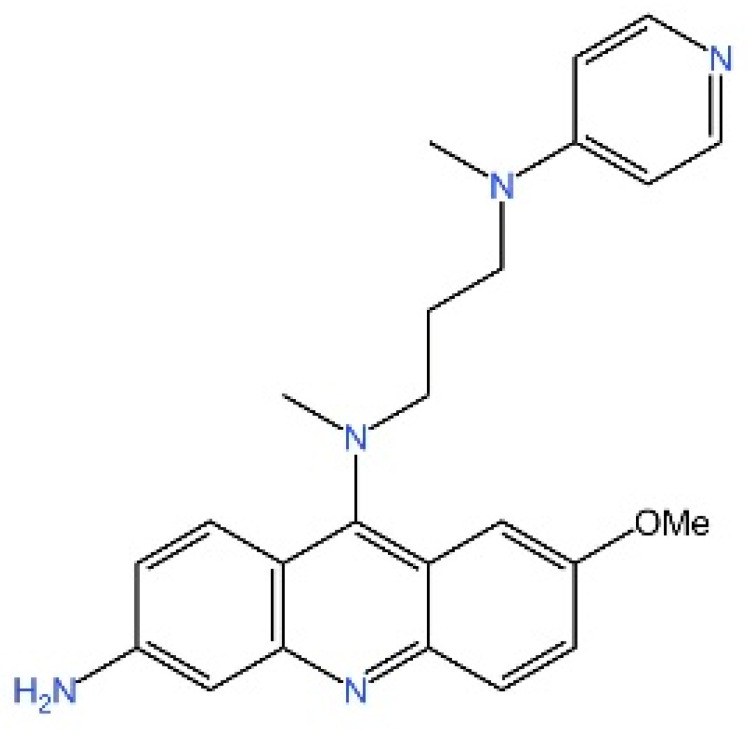	Inhibiting cGAMP synthesis in Trex1-deficient mice	[233]
Suramin	Competitive inhibitor	Inhibiting cGAS-DNA interaction (human)	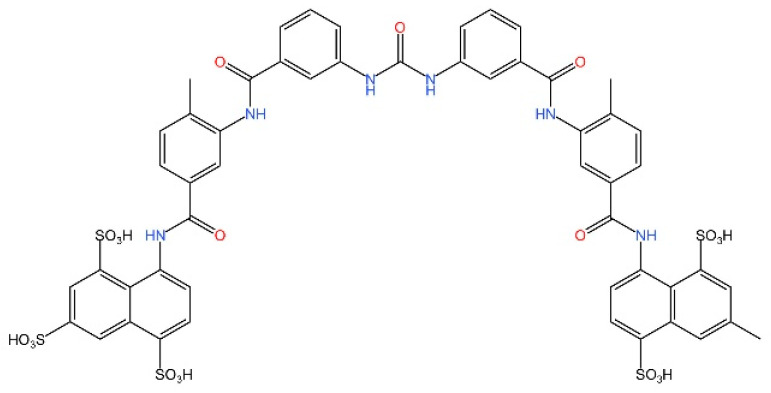	Reducing IFN-β pro-duction in THP-1 cells	[234]
4-sulfonic calix[6]	Competitive inhibitor	Inhibiting cGAS-DNA interaction/ Inhibiting catalytic site of cGAS (mouse)	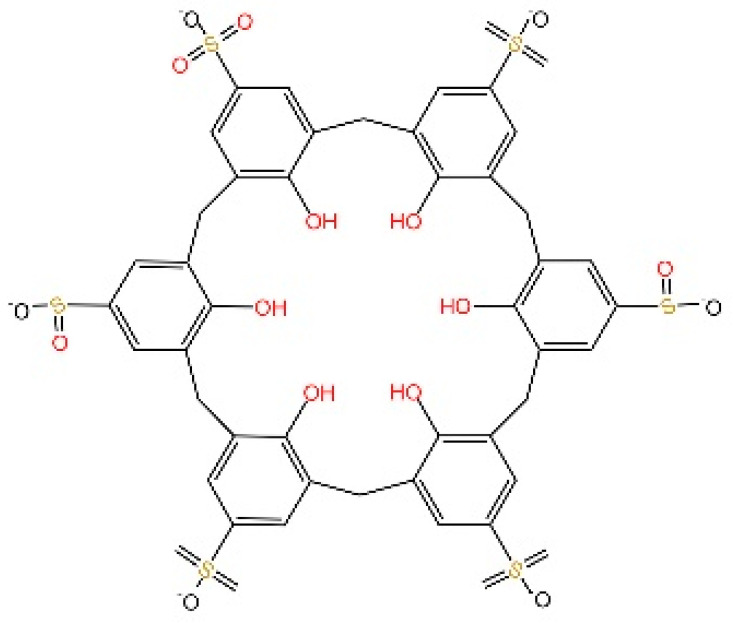	Preventing AIM2-dependent immunosuppression following stroke.	[235]
A151	Competitive inhibitor	Inhibiting cGAS-DNA interaction (human & mouse)	5′-TTAGGGTTAGGG TTAGGGTTAGGG-3′	Inhibiting type I interferon response in TREX1-deficient cells.	[261]
XQ2B	Competitive inhibitor	Inhibiting cGAS-DNA interaction (mouse)	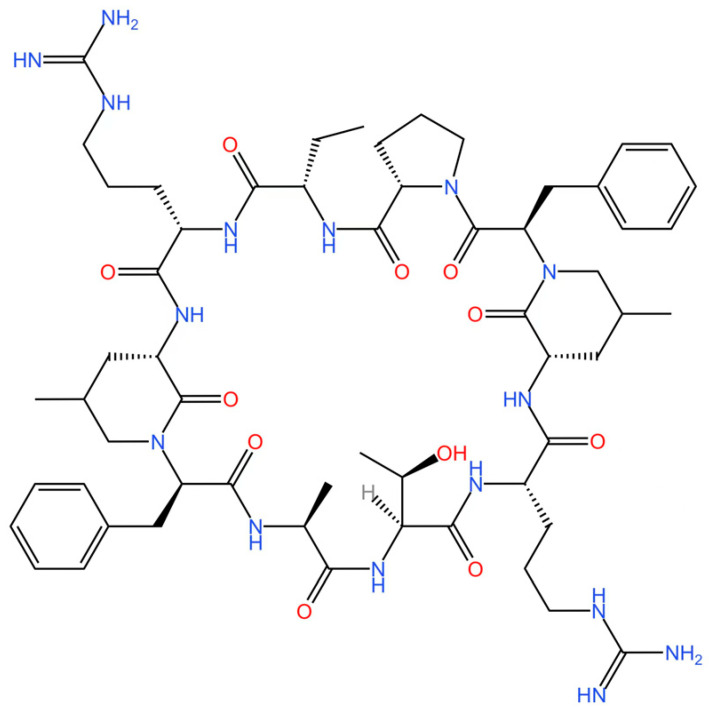	Inhibiting STING-mediated inflammation induced by Trex1 deficiency and in inhibiting IFN I production during HSV-1 infection.	[236].
STING antagonists targeting the CDN-binding site					
Compound **18**	cGAMP-competitive inhibitor	Inhibiting CDN-binding site of STING (human)	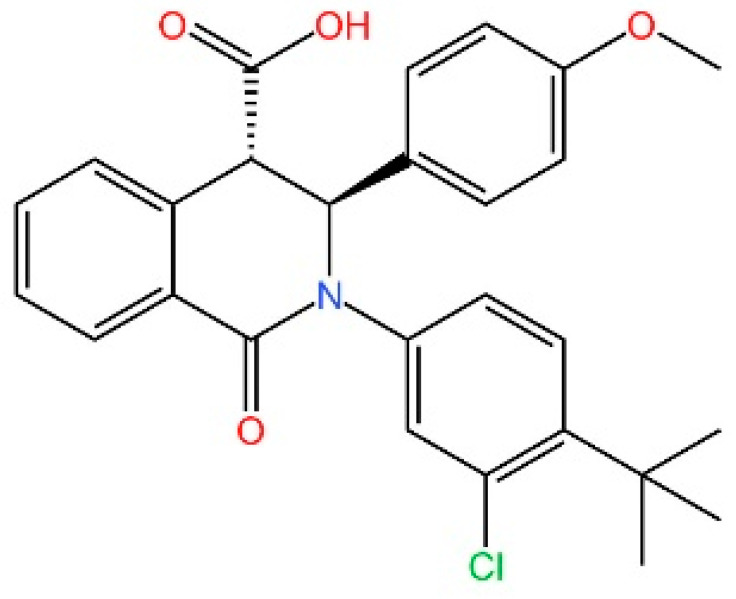	Inhibiting cGAMP-induced IFN-β secretion.	[239]
Astin C	cGAMP-competitive inhibitor	Inhibiting CDN-binding site of STING (mouse & human)	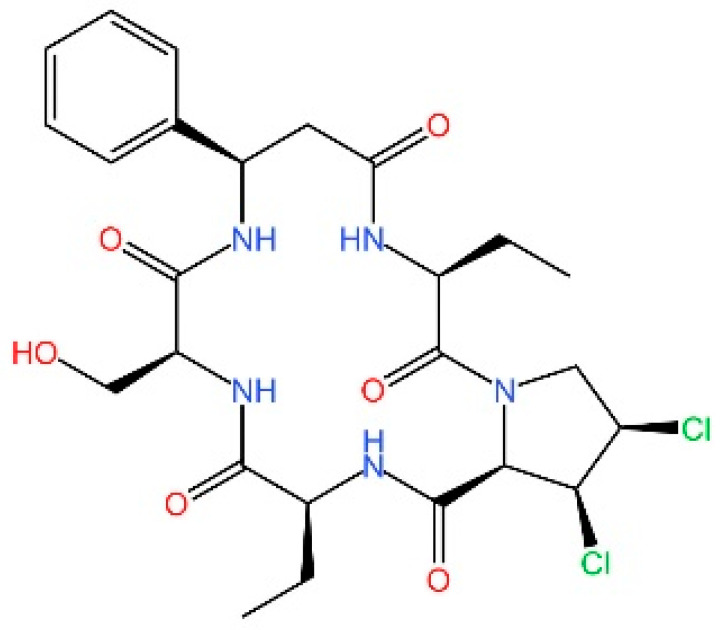	Attenuating the autoinflammatory responses in *Trex1*^−/−^ mouse autoimmune disease model.	[240]
SN-011	cGAMP-competitive inhibitor	cGAMP-competitive inhibitor (mouse & human)	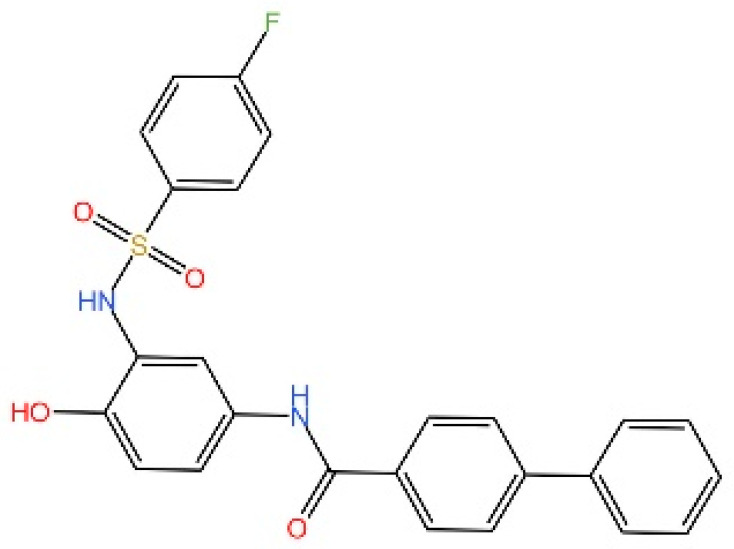	Suppressing Systemic Inflammation in *Trex1*^−/−^ mice.	[241]
Gelsevirine	cGAMP-competitive inhibitor	cGAMP-competitive inhibitor (human & mouse)	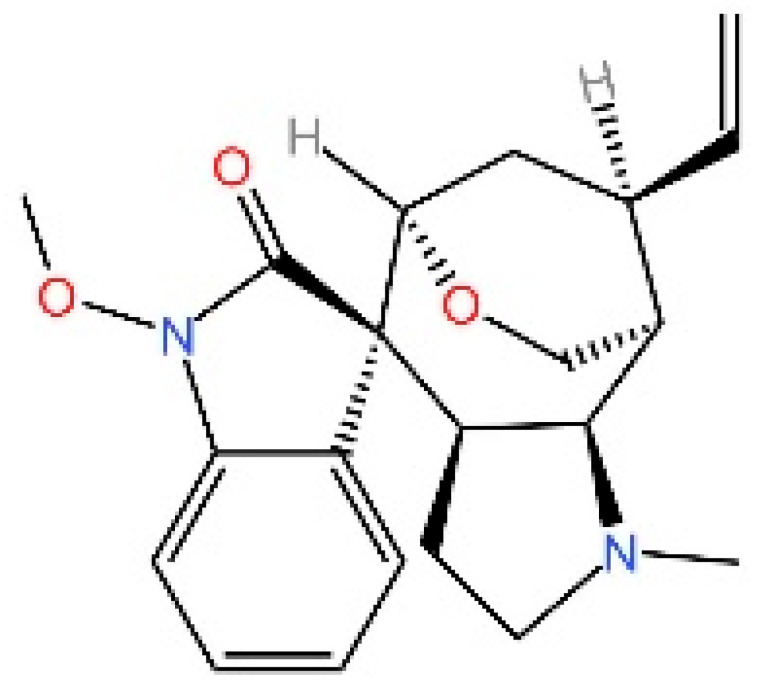	Mitigating STING-related inflammation in sepsis.	[242]
T0901317	SMPDL3A agonist	Degradation of cGAMP via LXR-SMPDL3A (mouse & human)	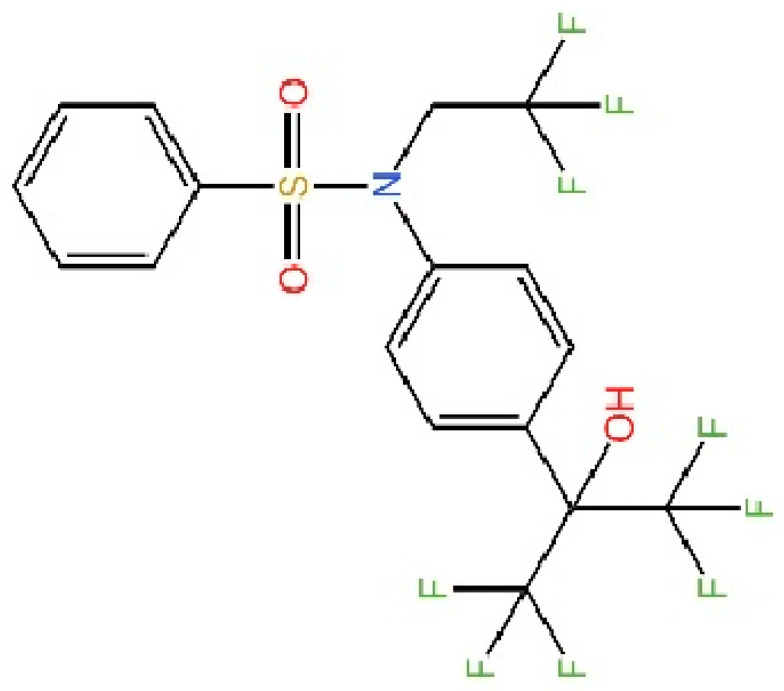	In the Smpdl3a^+/+^ mouse model, T0901317 induces an increase in HSV-1 viral load.	[243]
Anhydrotuberosin		cGAMP-competitive inhibitor (mouse & human)	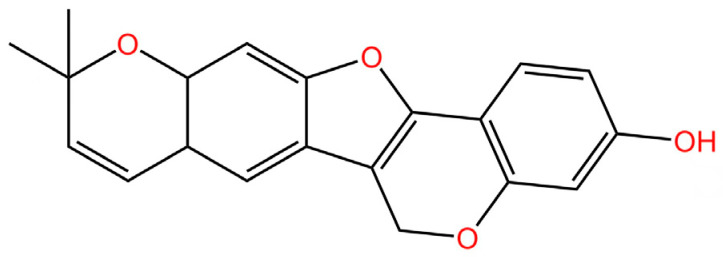	ATS prevents aberrant STING activation driven by SAVI-related mutations.	[244]
Targeting STING phosphorylation sites					
Meloxicam	No report	Inhibiting phosphorylation of STING (human & mouse)	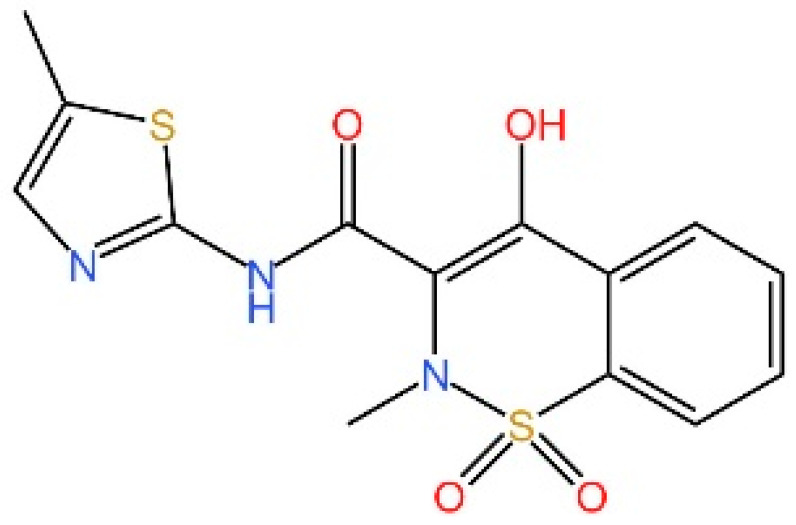	Promoting the survival in *Trex1*^−/−^ mouse model for Aicardi-Goutières syndrome	[246]
LH531	Covalently modified inhibitor	Inhibiting phosphorylation of STING (human)	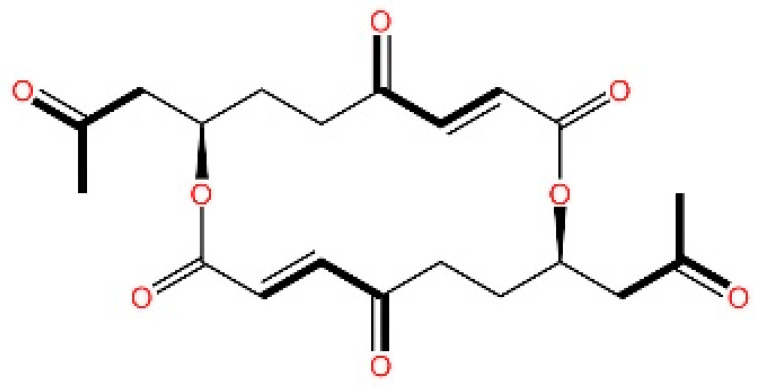	No report	[245]
LH519	Covalently modified inhibitor	Inhibiting phosphorylation of STING (human)	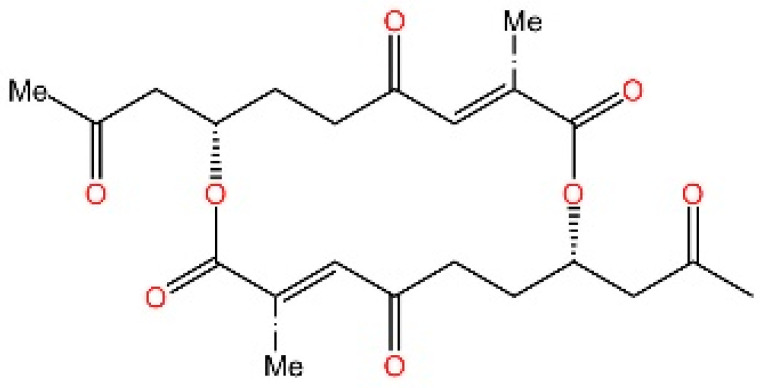	No report	[245]
4-OI	Covalently modified inhibitor	Inhibiting phosphorylation of STING (human & mouse)	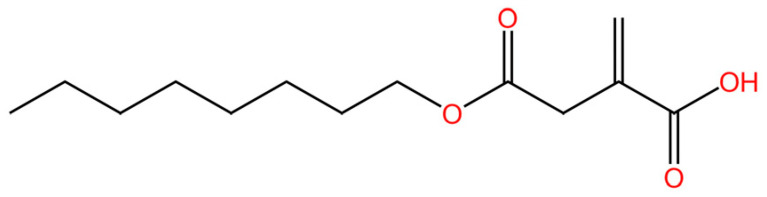	Alleviate sepsis	[247]
Targeting STING palmitoylation sites					
C-176	Covalently modified inhibitor	Inhibiting palmitoylation of STING (human & mouse)	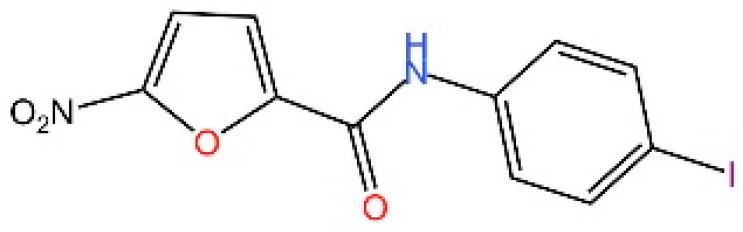	Reducing STING agonist mediated elevation of serum levels of type I IFNs and IL-6 in *Trex1*^−/−^ mice	[249]
C-178	Covalently modified inhibitor	Inhibiting palmitoylation of STING (human & mouse)	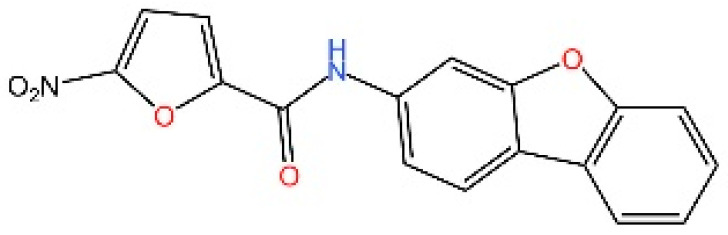	Suppressing the STING responses elicited by distinct activators	[249]
H151	Covalently modified inhibitor	Inhibiting palmitoylation of STING (human & mouse)	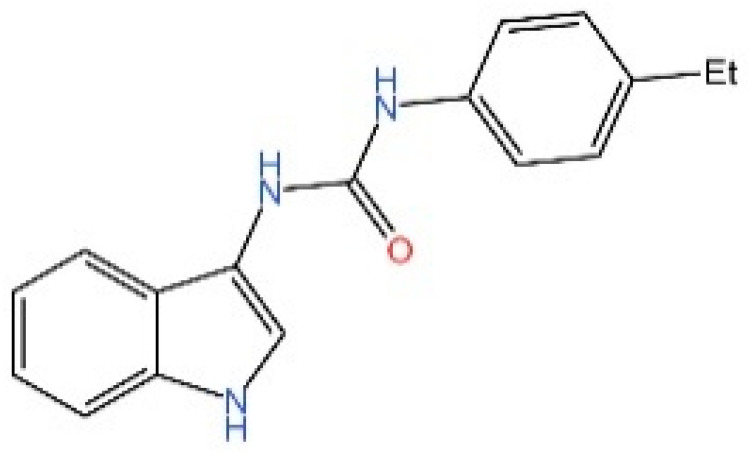	Reducing IFN-β levels in *Trex1*^−/−^ mice	[262]
NO2-cLA	Covalently modified inhibitor	Inhibiting palmitoylation of STING (human & mouse)	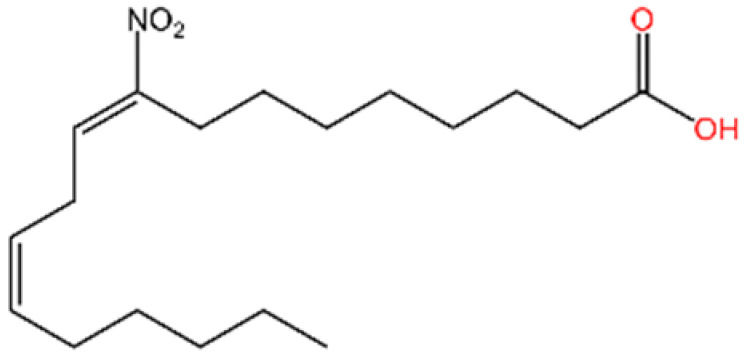	Reducing of type I IFNs in response to HSV-2 infection	[251]
NO2-OA	Covalently modified inhibitor	Inhibiting palmitoylation of STING (human & mouse)	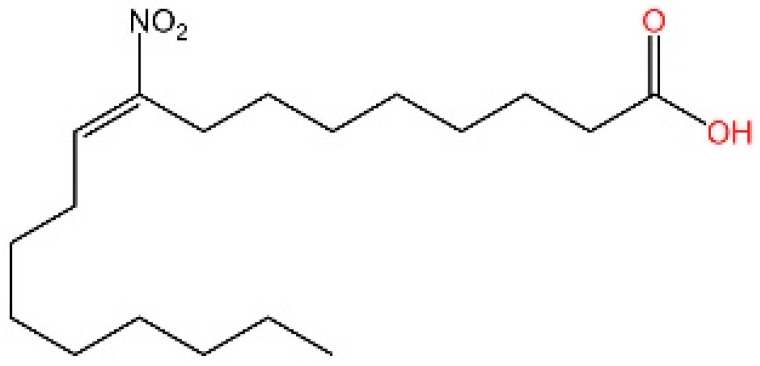	Inhibiting the increase in pTBK1 in fibroblasts from patients with SAVI	[251]
BPK-21	Covalently modified inhibitor	Inhibiting palmitoylation of STING (human & mouse)	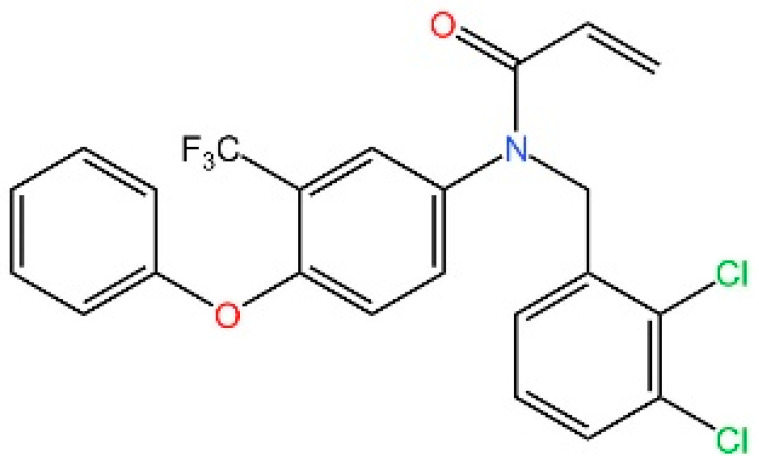	No report	[252]
BPK-25	Covalently modified inhibitor	Inhibiting palmitoylation of STING (human)	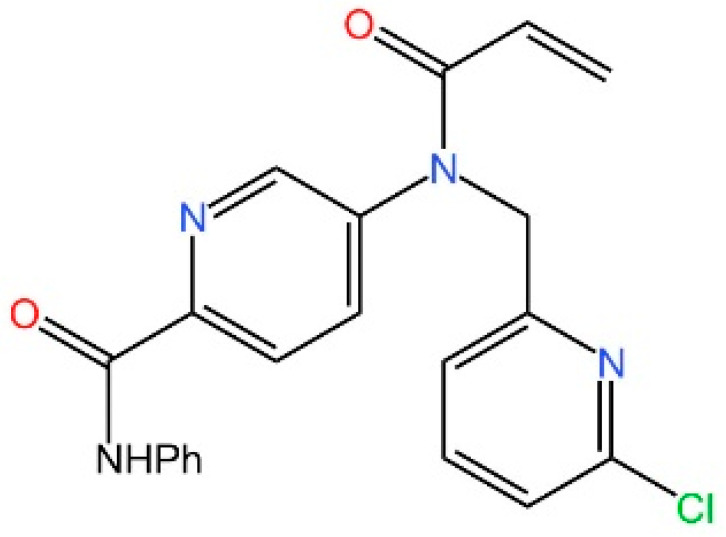	No report	[252]
4-OI	Covalently modified inhibitor	Inhibiting palmitoylation of STING (human & mouse)	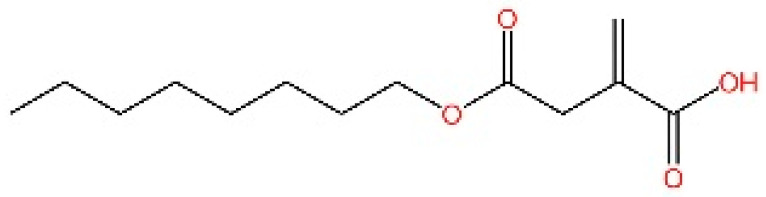	Restricting the cGAS-STING antiviral (HSV-1) immune response and inflammatory responses in *Trex1*^−/−^ Raw264.7 cells	[253]
NO2-cLA	Covalently modified inhibitor	Inhibiting palmitoylation of STING (human & mouse)	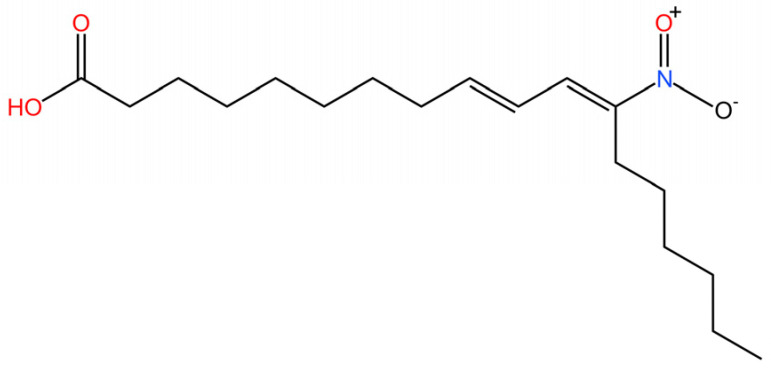	NO2-FAs inhibit release of IFN I from SAVI fibroblasts.	[251]
Targeting STING Cys292 sites					
LB224	Covalently modified inhibitor	Inhibiting modification of the Cys292 site of STING (human & mouse)	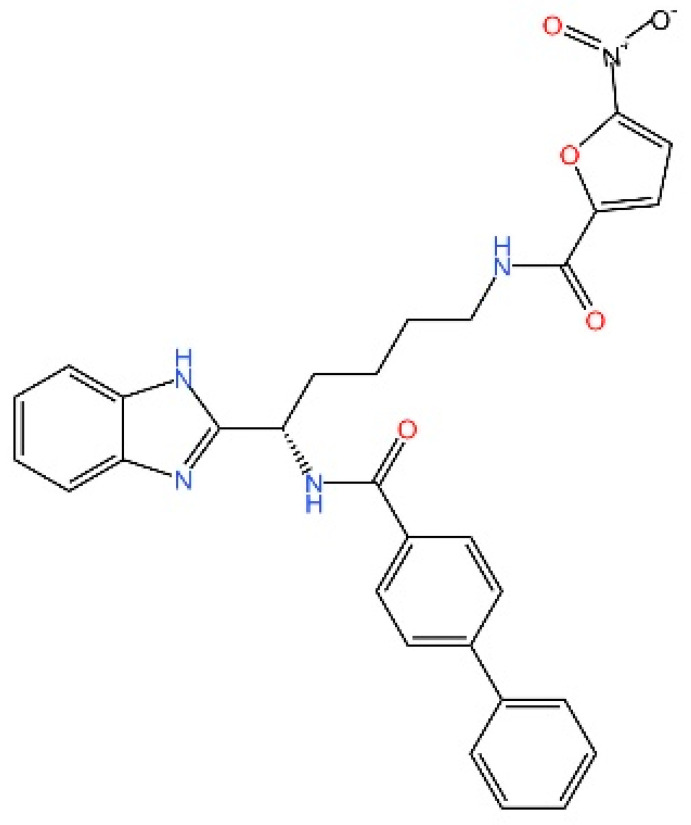	Reducing serum IFN-β and IL-6 after administration of diABZI in mice	[254]
Targeting STING degradation					
STING Degrader-1	Ubiquitin–proteasome system	Binding covalently to STING and E3 ligase (human & mouse)	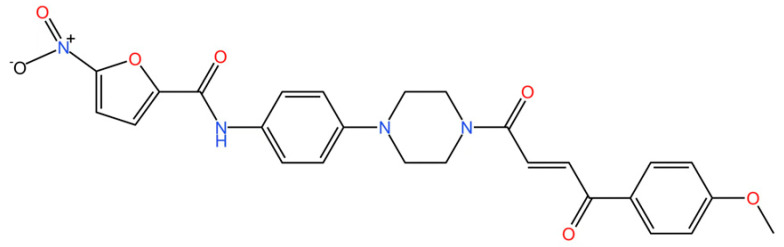	No report	[256]
SP23	Ubiquitin–proteasome system	Binding covalently to STING and E3 ligase (human & mouse)	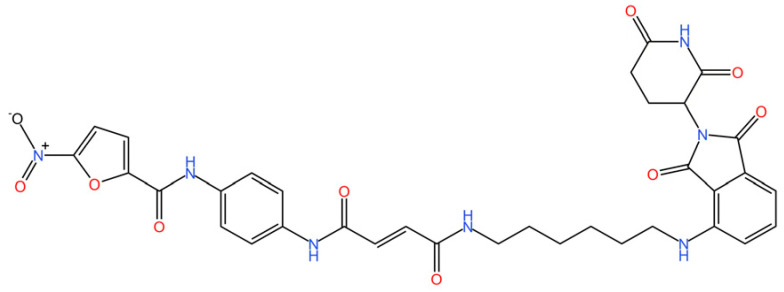	Alleviating cisplatin-induced acute kidney injury by inhibiting the STING signaling pathway.	[257]
UNC9036	Ubiquitin–proteasome system	Binding covalently to STING and E3 ligase (human & mouse)	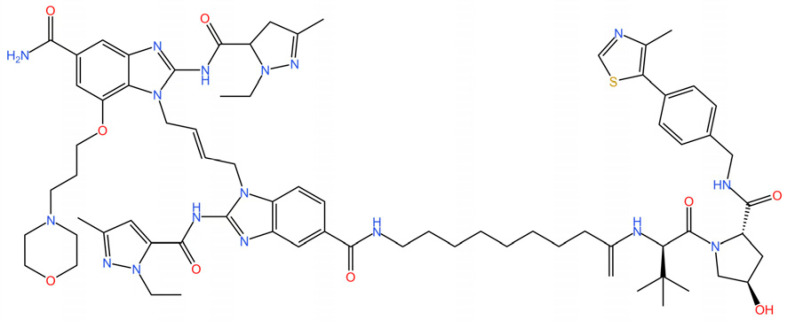	No report	[258]
Targeting STING trafficking					
4-phenylcinnamic acid	modulating STING trafficking	Competitive inhibitor	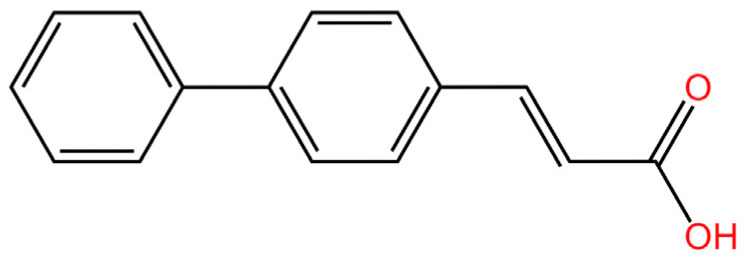	Significantly diminishing COPII-mediated vesicular transport of STING, it suppresses inflammatory signaling pathways.	[110,263]

## Data Availability

Not applicable.

## Abbreviations

The following abbreviations are used in this manuscript:

AC	Aortic Constriction
ACBD3	Acyl-CoA Binding Domain Protein 3
ACSL4	Acyl-coenzyme A (CoA) Synthetase Long-chain Family Member 4
ADAR1	Adenosine Deaminase Acting on RNA 1
AGS	Aicardi-Goutières Syndrome
AIM2	Absent in Melanoma 2
ALI	Acute Lung Injury
ALS	Amyotrophic Lateral Sclerosis
AMP	Adenosine Monophosphate
Ang II	Angiotensin II
AP	Acute Pancreatitis
APOE	Apolipoprotein E
ARDS	Acute Respiratory Distress Syndrome
ATM	Ataxia Telangiectasia Mutated
ATR	ATM and Rad3-Related
ATP	Adenosine Triphosphate
BSN	Bilateral Striatal Necrosis
CDN	Cyclic Dinucleotide
COP II	Coat Protein Complex II
CTT	C-Terminal Tail
CYLD	Cylindromatosis Lysine 63 Deubiquitinase
cGAMP	Cyclic GMP-AMP
cGAS	Cyclic GMP-AMP Synthase
COPA	Coatomer Subunit α
DAMP	Damage-Associated Molecular Pattern
DDX41	DEAD-Box Helicase 41
DCs	Dendritic Cells
DNA-PK	DNA-Dependent Protein Kinase
DNase	Deoxyribonuclease
DOT1L	Disruptor of Telomeric Silencing 1-Like
dsDNA	Double-Stranded DNA
DSS	Dextran Sulfate Sodium
eIF3f	Eukaryotic Translation Initiation Factor 3 Subunit F
ER	Endoplasmic Reticulum
ERGIC	ER-Golgi Intermediate Compartment
ESCRT	Endosomal Sorting Complex Required for Transport
FASN	Fatty Acid Synthase
GS	Gelsevirine
GTP	Guanosine Triphosphate
hnRNPA2B1	Heterogeneous Nuclear Ribonucleoprotein A2/B1
HSC	Hepatic Stellate Cell
HSV-1	Herpes Simplex Virus Type 1
HSV-2	Herpes Simplex Virus Type 2
ICAM-1	Intercellular adhesion molecule 1
IFI16	Interferon Gamma Inducible Protein 16
IFIH1	Interferon Induced with Helicase C Domain 1
IFN	Interferon
IFNAR1	Interferon Alpha/Beta Receptor 1
IFNAR2	Interferon Alpha/Beta Receptor 2
IKK	IκB Kinase
IL-1β	Interleukin-1 Beta
IL-6	Interleukin-6
IL-18	Interleukin-18
ILD	Interstitial Lung Disease
iNOS	Inducible Nitric Oxide Synthase
iRhom2	Inactive Rhomboid Protein 2
IRF3	Interferon Regulatory Factor 3
IRF9	Interferon Regulatory Factor 9
ISG	Interferon-Stimulated Gene
Jak1	Janus Kinase 1
JNK1	c-Jun N-Terminal Kinase 1
K48	Lysine 48-Linked Ubiquitination
LAMP1	Lysosomal Associated Membrane Protein 1
LCN2	Lipocalin 2
LSM11	U7 small nuclear RNA associated protein
LPS	Lipopolysaccharide
LXR	Liver X Receptor
MDA5	Melanoma Differentiation-Associated Protein 5
MEF	Mouse Embryonic Fibroblasts
mtDNA	Mitochondrial DNA
MXC	Meloxicam
MyD88	Myeloid Differentiation Primary Response 88
NAFLD	Non-Alcoholic Fatty Liver Disease
NEMO	NF-κB Essential Modulator
NET	Neutrophil Extracellular Trap
NF-κB	Nuclear Factor Kappa B
NLRP3	NLR Family Pyrin Domain Containing 3
NO2-cLA	Nitro-Conjugated Linoleic Acid
NO2-OA	Nitro-Oleic Acid
NPC1	Niemann-Pick Type C1
NSCLC	Non-Small Cell Lung Cancer
p-IRF3	Phosphorylated IRF3
p-tau	Phosphorylated Tau
PAMP	Pathogen-Associated Molecular Pattern
PBMC	Peripheral Blood Mononuclear Cell
PERK	Protein Kinase R-like ER Kinase
PIKK	Phosphoinositide-3-Kinase-Related Kinase
PLK1	Polo-Like Kinase 1
PM2.5	Particulate Matter ≤2.5 μm
PRRs	Pattern Recognition Receptors
PROTAC	Proteolysis-Targeting Chimera
PtdIns(3)P	phosphatidylinositol-3-phosphate
RIG-I	Retinoic Acid-Inducible Gene I
RLR	RIG-I-Like Receptor
RNF5	Ring Finger Protein 5
RNF90	Ring Finger Protein 90
RNU7-1	U7 Small Nuclear 1
ROS	Reactive Oxygen Species
SAVI	STING-Associated Vasculopathy with Onset in Infancy
SAMHD1	SAM and HD Domain Containing Protein 1
SCAP	SREBP Cleavage-Activating Protein
SLE	Systemic Lupus Erythematosus
SMPDL3A	Sphingomyelin Phosphodiesterase Acid-Like 3A
SREBP2	Sterol Regulatory Element-Binding Protein 2
STAT	Signal Transducer and Activator of Transcription
STEEP	STING ER Exit Protein
STIM1	Stromal Interaction Molecule 1
STING	Stimulator of Interferon Genes
TBK1	TANK-Binding Kinase 1
TCR	T Cell Receptor
TLR	Toll-Like Receptor
TNF-α	Tumor Necrosis Factor Alpha
TOLLIP	Toll-Interacting Protein
TREX1	Three Prime Repair Exonuclease 1
TRIF	TIR-Domain-Containing Adapter-Inducing Interferon-β
TRIM13	Tripartite Motif Containing 13
TRIM21	Tripartite Motif Containing 21
TRIM29	Tripartite Motif Containing 29
TRIM30α	Tripartite Motif Containing 30 Alpha
Tyk2	Tyrosine Kinase 2
USP18	Ubiquitin-Specific Peptidase 18
USP20	Ubiquitin-Specific Peptidase 20
V155M	Valine 155 to Methionine Mutation
WDR5	WD Repeat Domain 5
XBP1	X-Box Binding Protein 1
YY1	Yin Yang 1
